# SCMR expert consensus statement for cardiovascular magnetic resonance of acquired and non-structural pediatric heart disease

**DOI:** 10.1186/s12968-022-00873-1

**Published:** 2022-07-21

**Authors:** Adam L. Dorfman, Tal Geva, Margaret M. Samyn, Gerald Greil, Rajesh Krishnamurthy, Daniel Messroghli, Pierluigi Festa, Aurelio Secinaro, Brian Soriano, Andrew Taylor, Michael D. Taylor, René M. Botnar, Wyman W. Lai

**Affiliations:** 1 Department of Pediatrics, Division of Pediatric Cardiology, University of Michigan C.S. Mott Children’s Hospital, 1540 E. Medical Center Drive, Ann Arbor, MI 48109 USA; 2 Department of Cardiology, Boston Children’s Hospital, 300 Longwood Ave, Boston, MA 02115 USA; 3 Department of Pediatrics, Division of Pediatric Cardiology, Medical College of Wisconsin/Herma Heart Institute, Children’s Wisconsin, Milwaukee, WI 53226 USA; 4 Department of Pediatrics, Division of Pediatric Cardiology, University of Texas Southwestern Medical Center, Dallas, TX 75235 USA; 5 Department of Radiology, Nationwide Children’s Hospital, 700 Children’s Dr. E4A, Columbus, OH 43205 USA; 6 Department of Internal Medicine-Cardiology, Deutsches Herzzentrum Berlin and Charité-University Medicine Berlin, Berlin, Germany; 7 Department of Cardiology, Fondazione Toscana G. Monasterio, Massa, Italy; 8 Advanced Cardiothoracic Imaging Unit, Department of Imaging, Bambino Gesù Children’s Hospital IRCCS, Rome, Italy; 9 Department of Pediatrics, Division of Pediatric Cardiology, Seattle Children’s Hospital, 4800 Sand Point Way NE, Seattle, WA 98105 USA; 10 Department of Cardiovascular Imaging, Great Ormond Street Hospital for Sick Children, University College London, London, UK; 11 Department of Pediatrics, Division of Pediatric Cardiology, Cincinnati Children’s Hospital, 3333 Burnet Ave #2129, Cincinnati, OH 45229 USA; 12 School of Biomedical Engineering and Imaging Sciences, King’s College London, London, UK; 13 CHOC Children’s, 1201 W. La Veta Avenue, Orange, CA 92868 USA

**Keywords:** Cardiovascular magnetic resonance, Pediatric heart disease, Guidelines, Children

## Abstract

Cardiovascular magnetic resonance (CMR) is widely used for diagnostic imaging in the pediatric population. In addition to structural congenital heart disease (CHD), for which published guidelines are available, CMR is also performed for non-structural pediatric heart disease, for which guidelines are not available. This article provides guidelines for the performance and reporting of CMR in the pediatric population for non-structural (“non-congenital”) heart disease, including cardiomyopathies, myocarditis, Kawasaki disease and systemic vasculitides, cardiac tumors, pericardial disease, pulmonary hypertension, heart transplant, and aortopathies. Given important differences in disease pathophysiology and clinical manifestations as well as unique technical challenges related to body size, heart rate, and sedation needs, these guidelines focus on optimization of the CMR examination in infants and children compared to adults. Disease states are discussed, including the goals of CMR examination, disease-specific protocols, and limitations and pitfalls, as well as newer techniques that remain under development.

## Background

Cardiovascular magnetic resonance imaging (CMR) has rapidly gained acceptance and is now established as an essential imaging modality for the pediatric population. A large and unique component of CMR use in children is for imaging structural congenital heart disease (CHD), for which a guidelines document has been published [1]. Traditionally, hereditary cardiomyopathies (e.g., hypertrophic cardiomyopathy) are not categorized in this CHD group, even if technically they are characterized by abnormalities of myocardial structure that are present at birth. There are also patients with acquired, non-CHD (e.g., Kawasaki Disease) that may become manifest in childhood. For these pediatric patients, CMR represents an equally important diagnostic modality as for those with CHD. Given the unique technical and diagnostic challenges of CMR evaluation of infants and children with heart disease, there has been interest in creating a CMR guidelines document for imaging pediatric patients with non-CHD. In this document the term “non-CHD” is used to refer to this group of diseases as distinct from structural CHD lesions that are part of the previous guidelines document.

Several of the diseases discussed in this document have equivalents in the adult population, for whom standardized CMR protocols were recently updated [2]. However, the clinical presentation, disease course, and pathophysiology of these conditions are often substantially dissimilar in the pediatric population and require different imaging strategies. Further, small body size and fast heart rate in infants and young children require adjustments of CMR protocols to optimize image quality and facilitate accurate diagnosis. Additionally, the normative data available for ventricular volume, mass and function in pediatric patients are not as robust as in the adult population, as only limited datasets of normal values for children are available in the literature [3–8]. Adult-based data cannot be directly extrapolated to the pediatric population based on body size. For example, the normal left ventricular (LV) end-diastolic volume indexed to body surface area (LVEDVI) for a small child is not the same as the normal LVEDVI for an adult [9]. The lack of universal normative data increases the difficulty of accurately interpreting and reporting pediatric studies.

There is a strong desire to standardize approaches to CMR for non-CHD in the pediatric population. There is potential for clinical benefit when standardized protocols for children are used as a best practice approach. Standardized imaging protocols in CMR for acquired and non-CHD in children will improve quality and reduce practice variability. There also continues to be growing interest in a multi-center, collaborative approach to CMR-based clinical research, including the collection of normative data across large and diverse populations of children, which would be facilitated by standardized protocols.

## Methodology

After the initial conception of this manuscript, the expert panel was formed with guidance from the Society for Cardiovascular Magnetic Resonance (SCMR) Executive Committee and approved by the SCMR Board of Trustees. Care was taken to include representation of cardiologists, radiologists and basic scientists with recognized expertise in pediatric and congenital cardiovascular magnetic resonance. Members were also chosen from both North America and Europe to represent the large footprint of SCMR. After initial meetings generated the list of cardiovascular disease states to be included in this work and a framework for the discussion, each disease or lesion was assigned a primary and a secondary writer to collaborate on that section. The aim was to focus on practical clinical use of CMR. The individual pieces were then collated and edited for consistency to create the final document. The document was then reviewed by an external group of CMR and disease experts, revised, and ultimately approved by the SCMR Publications Committee and Board of Trustees.

### Special considerations for imaging pediatric patients

There are unique aspects to the performance of CMR studies in children that can pose difficulties. The size of young children, for example, presents challenges for optimizing CMR sequence parameters. In small infants, the signal-to-noise ratio (SNR) is low overall. For adequate spatial resolution in some pediatric cases a decreased voxel size may be optimal or even necessary, but this leads to a further reduction of SNR. Options for improving SNR for small children include increased signal averages, at the cost of longer scan time, or increasing repetition time (TR) for spin echo sequences. For the latter, a sequence that typically has a TR of 2 cardiac cycles can be increased to 3 cardiac cycles, resulting in improved SNR but increasing scan time and altering the T1 weighting. SNR modifications usually require increased scan time to maintain comparable spatial resolution. Note that increased scan time with breath holding under anesthesia may or may not be tolerated depending on the physiology of an individual patient; issues around sedation and anesthesia are discussed below.

Higher heart rates can pose a number of difficulties for temporal resolution, for having adequate time for signal recovery within certain sequences, or time for the pulse sequences themselves. For example, performing long inversion time (600 ms) late gadolinium enhancement (LGE) sequence for thrombus detection [10] in a child with a heart rate of 120 bpm (cardiac cycle length of 500 ms) requires specific modifications. One option in this situation is to change parameters so that the scanner treats the heart rate as half of the actual value, doubling scan time but allowing adequate time for inversion recovery. As heart rate increases, it is the length of diastole rather than systole that decreases. Avoiding cardiac motion artifact for still images typically acquired in diastole requires decreased image acquisition time per cardiac cycle, at the cost of prolonging overall scan time. For cine images, the temporal resolution must be shorter; this is typically achieved by decreasing the acquired k-space segments per cardiac cycle. Most of these heart rate-based modifications minimally increase overall scan time, as the fewer acquisitions per cardiac cycle are balanced by a greater number of cardiac cycles per second.

Parametric imaging techniques (i.e., T1 and T2 mapping) have gradually migrated from research applications to the clinical realm over several years. The writing group recognizes that these techniques may not be available or accessible at all centers at the time of this writing, but clinical use has increased; thus, these sequences are included in the recommendations for some of the following protocols, as appropriate. When parametric imaging is not available, the protocols in this document are still recommended without those sequences, but we encourage all centers to gain experience with these pulse sequences and establish local normative data [11, 12].

The basics of parametric imaging are the same in children as in adults and have been well described in the literature. However, a number of factors can make these techniques difficult in a pediatric population. Automated motion correction should be applied as available, but there is not currently a widely clinically available method for parametric imaging during free breathing. This poses significant difficulty for young children who may be unable to breath hold. In most cases, the clinical benefit of these data do not warrant general anesthesia with endotracheal intubation for breath holding if not otherwise necessary for the study. Higher heart rates may preclude adequate relaxation time, compromising data accuracy. A scheme to address this issue involves a time-based rather than heartbeat-based recovery time to optimize T1 recovery, independent of heart rate. Finally, there are limited published data for the use of parametric mapping in children that are linked to outcomes. With time, wider adoption of the use of parametric imaging sequences will generate better data for understanding these CMR findings and may help translate them into clinical management decisions.

### Sedation and general anesthesia

The requirement of deep sedation or general anesthesia for young children undergoing a CMR study necessitates decisions involving appropriateness, scheduling, examination performance, and the interpretation of findings when sedation or anesthesia protocols vary. A discussion of the appropriateness and timing of CMR is beyond the scope of this document. There are risks inherent with the use of sedatives or anesthetic agents that impact hemodynamic status and may result in hypotension or hypoxemia in some patients [13]. However, CMR under general anesthesia is safe when provided by experienced pediatric anesthesiologists [14]. Children under 6 years of age undergoing CMR routinely require sedation. At any age, the decision to use sedation or general anesthesia must be made after consideration of the risks, the value of the clinical information to be obtained, and alternative imaging modalities. Institutional guidelines for sedation and general anesthesia must be adhered to, and informed consent should be obtained whenever appropriate [15, 16]. Alternative techniques, such as involvement of the Child Life team, can help some children to successfully complete a CMR without sedation or anesthesia [17].

Hypertrophic cardiomyopathy (HCM) and pulmonary hypertension (PH) are disease states associated with higher risks of adverse events with anesthesia, and data are limited on the safety of CMR under sedation or anesthesia for these patients. The use of some sedative agents and inhaled anesthetics can have a profound impact on myocardial function and the cardiovascular system [18], and use of oxygen and positive pressure ventilation can impact on some flow measurements [19]. If the indications for a CMR study are primarily for assessing physiology rather than anatomy, those alterations in physiology should be considered when interpreting the data. Pre-procedural discussion between the CMR physician and the anesthesiologist can help guide decision making for the necessity of endotracheal intubation for breath holding and for the choice of sedative agents that may have specific hemodynamic effects. This communication is also important to discuss the impact of potential management changes during the case, such as volume boluses or changes in vasoactive medications, on CMR physiologic measurements.

Patients being assessed for shunt lesions (i.e., determination of pulmonary to systemic (Qp:Qs) flow ratio) should be maintained at as close to baseline hemodynamics as possible during phase contrast imaging. Importantly, this includes being maintained on room air if possible. It is similarly important to maintain end-tidal carbon dioxide levels in the normal range when studying patients with potential shunts or PH. Even relatively minor issues such as ensuring appropriate NPO (*nil per os*: no food or drink prior to sedation) status may require planning for a morning scan to avoid extended daytime NPO status for a study scheduled later in the day. Coordination between the CMR service and the pediatric anesthesia service, which ideally has focused expertise with pediatric cardiac patients, is crucial [13]. We strongly recommend that the physician responsible for monitoring and interpreting the CMR exam should not be responsible for monitoring sedation for the patient as well.

### CMR in specific disease states

#### Dilated cardiomyopathy and post-chemotherapy cardiomyopathy

Dilated cardiomyopathy (DCM) is the most common form of cardiomyopathy and cause of heart transplantation in children. The estimated incidence of pediatric cardiomyopathy based on presentation to a pediatric cardiologist (as opposed to genotype positivity) is 1.13 cases per 100,000 children, with DCM accounting for 51% of that total [20]. Most cases are idiopathic in origin (65%), and the most common known causes are myocarditis and neuromuscular disease [21]. Cardiomyopathy is also seen in the growing population of survivors of childhood cancer. In these patients, cardiovascular disease is the leading cause of non-cancer related morbidity and mortality [22].

CMR studies for pediatric DCM have focused on the early detection of myocardial involvement in children at high risk for developing ventricular dysfunction, such as muscular dystrophy patients [23–25] and pediatric cancer survivors exposed to anthracyclines and radiation [26, 27]. Myocardial fibrosis demonstrated by LGE was seen in association with ventricular dysfunction in the DCM or muscular dystrophy group [28–30], but not in the pediatric cancer survivor group [27, 31]. In a study of long-term survivors of childhood leukemia, LGE was associated with LV and right ventricular (RV) diastolic dysfunction [32].

#### Goals of examination

CMR is an established technique for the assessment of both LV and RV volumes and regional/global systolic function, and can be used to determine the etiology of ventricular dysfunction in dilated and other cardiomyopathies [33]. In adult patients with DCM, CMR studies are indicated to differentiate ischemic versus non-ischemic etiologies; determine ventricular size, function, and mass; detect intracardiac thrombus; and assess for LGE as a marker of fibrosis and for prognostic stratification [34, 35]. Children with non-ischemic DCM may have a non-coronary pattern of LGE with patchy or longitudinal mid-wall enhancement, subepicardial, or diffuse subendocardial distribution, but LGE is found less commonly than in adults with this disease [36, 37]. If in question, anatomic anomalies of the coronary arteries should be excluded during the first CMR investigation of children with newly diagnosed DCM.

The recommended sequences for assessment of DCM and post-chemotherapy cardiomyopathy are summarized in Table 1. Ventricular volume assessment with cine CMR (balanced steady state free precession (bSSFP)) has been standardized for both adults and children [1, 38, 39]. In the early stages of DCM, inflammation sequences (T2 signal intensity ratio and early gadolinium enhancement) are useful for distinguishing DCM from acute myocarditis [40]. This will be addressed further in the section on myocarditis. Using phase contrast flow sequences, Rosales et al. demonstrated abnormal indices of LV diastolic function in mostly adult patients with muscular dystrophy [41]. In pediatric cancer survivors, Ylanen et al. showed RV systolic dysfunction and elevated end-systolic volume [42], and using left atrial (LA) volume measurements, de Ville de Goyet et al. found evidence of LV diastolic dysfunction [26].

**Table 1:** Dilated and post-chemotherapy cardiomyopathy

Sequence	Imaging plane	Indication
Standard imaging		
Cine bSSFP	Short-axis stack	LV volumes, mass, and EF RV volumes, mass (short axis only), and EF Regional wall motion Intracardiac thrombus
	LV long axis views	
	Axial stack	RV volumes, and EF Intracardiac thrombus
Late gadolinium enhancement	Short-axis stack LV long-axis views RV-specific views	Focal fibrosis Prognostic stratification
Additional case-specific or comprehensive imaging		
Cine bSSFP	Short-axis or axial stack	LA volume
T2 signal intensity ratio	Short-axis stack LV long-axis views RV-specific views	Inflammation (edema)
Early gadolinium enhancement	Short-axis stack LV long-axis views RV-specific views	Inflammation (hyperemia)
T1-mapping pre- and post-gadolinium contrast	Short-axis view LV 4-chamber view	Inflammation Diffuse myocardial fibrosis Extracellular volume
Phase contrast flow mapping	Transmitral flow plane	LV diastolic function
Myocardial tagging	Short-axis views LV 4-chamber view	Myocardial strain (Alternative to use feature tracking for post-processing cine bSSFP sequences)
First-pass perfusion	Short-axis views LV 4-chamber view	Myocardial perfusion

*EF* ejection fraction, *LA* left atrial, *LV* left ventricular, *RV* right ventricular, *bSSFP* Balanced steady state free precession

#### Limitations and pitfalls

The role of CMR for pediatric patients either with or at risk for DCM has yet to be firmly established. Many children have adequate windows for transthoracic echocardiographic (TTE) evaluation of ventricular size and function, including deformation imaging and LA volume measurement. Cases of ischemic DCM, perhaps the most common indication in adults, are rare in children, and stress imaging is infrequently indicated. Currently, the primary clinical benefit of CMR, as discussed further below, may be its ability to distinguish inflammatory causes of DCM, i.e., myocarditis, from other chronic conditions. The diagnosis of mid-wall LGE is technically challenging, because of the thin myocardial walls in children. In addition, mid-wall fibrosis is generally considered a nonspecific finding, and its clinical impact in the care of pediatric DCM requires further investigation.

#### Newer techniques

Newer techniques have been used to determine early markers for ventricular dysfunction. Reduced global circumferential strain (GCS) can be seen with CMR feature tracking (FT) or with tagged gradient echo cine sequences in patients with muscular dystrophy [23, 25, 43] and in pediatric cancer survivors [27, 44]. In addition, T1 mapping and extracellular volume fraction (ECV) measurements were found to possibly represent diffuse fibrosis in pediatric cancer survivors with normal LV ejection fraction (LVEF) [27, 45]. In Duchenne muscular dystrophy carriers, minor changes in extracellular volume have been demonstrated with T1-mapping with modified Look-Locker inversion recovery (MOLLI) [46]. First-pass perfusion defects have also been noted in pediatric cancer survivors [26].

### Left ventricular non-compaction

**Fig. 1:**
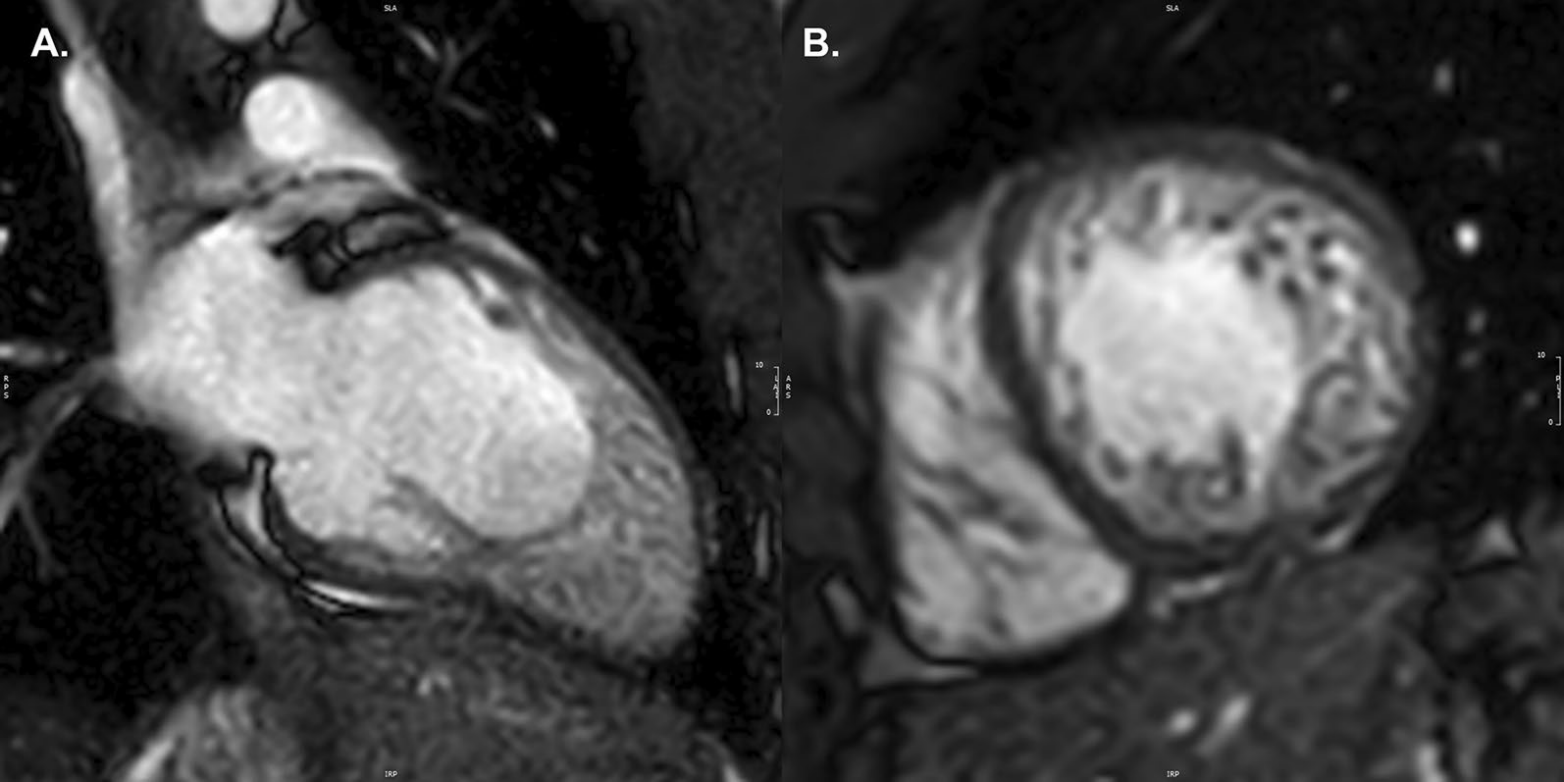
Left ventricular non-compaction. Vertical long axis (**A**) and mid-ventricular short axis (**B**) images of a patient with left ventricular non-compaction. Note the wide area of non-compact myocardium relative to the thin, compact wall

LV non-compaction (LVNC) is characterized by a thin compacted epicardial layer and a thick endocardial layer with prominent trabeculations and deep recesses [47] (Fig. 1A, B). Data derived from a large, adult-based cohort (the Multi-Ethnic Study of Atherosclerosis (MESA Study)) showed that more extensive non-compacted myocardium did not predict clinically significant LV enlargement or LV systolic dysfunction over a decade of follow-up. Thus, with a low pre-test probability for LVNC cardiomyopathy, these findings may not represent a pathologic condition and regular imaging follow-up may be unnecessary [48]. TTE remains the “gold standard” for the diagnosis of LVNC with three key echocardiographic criteria published to date [49], although there is still no universally accepted definition. The most well accepted echocardiographic criterion, based on data from an adult population, defines LVNC as likely with a ratio of non-compacted (NC) to compacted (C) myocardium (from LV *end-systolic* parasternal short axis images) of greater than 2.0 [50]. Of note, interobserver agreement for this measurement in children has been shown to be low [51]. CMR may add value in characterizing “concerning” LV trabeculations (indicative of LVNC) versus minor trabeculations that may be of no clinical significance (Table 2), particularly when echocardiography fails to visualize all ventricular segments. The importance of accurate assessment of LV size and function is emphasized by pediatric data showing the association of systolic dysfunction by TTE with death or transplantation in this disease [52].

**Table 2:** Left ventricular non-compaction

Sequence	Imaging plane	Indication
Standard imaging		
Cine bSSFP	Short-axis stack	LV volumes, mass, and EF RV volumes, mass, and EF Calculation of NC:C ratio (as noted in text)
	LV long-axis views	
Additional case-specific or comprehensive imaging		
T1-mapping pre- and post-gadolinium contrast	Short-axis view LV 4-chamber view	Fibrosis Extracellular volume
Late gadolinium enhancement	Short-axis stack LV long-axis views RV-specific views	Fibrosis
First-pass perfusion	Short-axis views LV 4-chamber view	Myocardial perfusion

*EF* ejection fraction, *LV* left ventricular, *RV* right ventricular, *bSSFP* balanced steady state free precession

### Goals of examination for LVNC

The goals of CMR for LVNC are to accurately measure LV size and LVEF, and to clearly image the LV myocardium in all segments from 2-chamber, 3-chamber, 4-chamber and short-axis imaging planes. CMR methods for assessing the non-compact:compact (NC:C) myocardial ratio were established in adult populations. More recently, studies have established that extent of LVNC and LGE in this disease is similar between children or adolescents and young adults [53].

The method of Petersen et al. [54], uses bSSFP cine imaging of 3 LV long-axis *diastolic* images to identify the myocardial segment with the most pronounced trabeculations. The compacted myocardial length is measured from the LV epicardium to the trough of a trabeculation, while the global myocardial length is from the epicardium to the peak of a trabeculation. The non-compacted myocardial length is calculated as the difference. The NC:C ratio can be determined for any LV segment with a ratio of greater than 2.3:1 distinguishing pathologic LVNC [54]. A different method published by Jacquier and colleagues relies on planimetry of a short axis bSSFP cine stack in diastole. The compact myocardium is contoured, including the papillary muscles. This is compared with a secondary set of short axis tracings that allocate all fine trabeculations to the myocardium, giving global mass. Thus, trabeculated mass, also known as non-compacted LV mass, is equal to Global LV mass minus compacted LV mass [55]. A non-compacted LV myocardial mass greater than 20% of the total LV mass is suggestive of LVNC.

In the pediatric population, a study has established the presence of LGE as a predictor of adverse events [56], although this study was limited by low patient numbers.

#### Limitations and pitfalls

As with other imaging modalities in LVNC, it is not possible to compare CMR findings to a true “gold standard” for diagnosis of LVNC. The diagnostic criteria of Petersen et al. [54] listed above is widely used to make this diagnosis, but the more recent MESA data showing lack of prognostic value for more extensive non-compact myocardium raises important questions. More robust outcomes-based data for CMR findings in LVNC will be important in the future.

#### Newer techniques for LVNC

T1-mapping [57] and LGE imaging techniques have been shown to identify patients with myocardial fibrosis in the setting of LVNC. These patients may be at higher risk for diminished LV systolic function [58] in adult data, or cardiovascular death and transplantation [56] in children. Additional tissue characterization studies (looking at high-intensity endocardial T2 signals [59] and subendocardial perfusion defects) may enhance understanding of the LVNC patient [60], but data are quite limited in the pediatric population. CMR strain imaging is a promising technique for assessing myocardial function in LVNC, with longitudinal and circumferential indices diminished in affected mid-ventricular and apical regions [61]. Children and adolescents with LVNC and normal LVEF have been characterized as having decreased CMR-derived LV strain parameters [53]. Patients with LVNC and decreased LVEF may be at risk of thrombus formation within the LV trabeculae. Published data on use of CMR to detect thrombus in this condition are restricted to isolated case reports in adults, but LGE with long inversion time of 600 ms can be considered in the patient thought to be at high risk for thrombus [10].

### Arrhythmogenic right ventricular cardiomyopathy

Arrhythmogenic right ventricular cardiomyopathy (ARVC) is characterized by fibro-fatty replacement of myocardium and development of ventricular arrhythmias. This diagnosis is now sometimes referred to as arrhythmogenic cardiomyopathy (AC), as involvement of the LV has been increasingly recognized. The gold standard for the diagnosis of ARVC is based on histology. Diagnosis of ARVC may be made in part by CMR, which is included in the 2010 American Heart Association ARVC Taskforce Criteria [62, 63]. ARVC is diagnosed if a patient has either 2 major criteria; 1 major and 2 minor criteria; or 4 minor criteria. The CMR-specific criteria are as follows:

Major

- Regional RV akinesia or dyskinesia or dyssynchronous RV contraction *and* 1 of the following:
  - RV end-diastolic volume/body surface area (BSA) ≥ 110 mL/m^2^ (male) or ≥ 100 mL/m^2^ (female)
  - RV ejection fraction (RVEF) ≤ 40%
    Minor
- Regional RV akinesia or dyskinesia or dyssynchronous RV contraction *and* 1 of the following:
  - RV end-diastolic volume/BSA ≥ 100 to < 110 mL/m^2^ (male) or ≥ 90 to < 100 mL/m^2^ (female)
  - RV ejection fraction (RVEF) > 40 to ≤ 45%

The importance of CMR in the context of the 2010 revised Task Force Criteria for the diagnosis of ARVC in children and adolescents was established in a study of 142 pediatric patients, which also showed the limited value of qualitative assessment of myocardial fat infiltration in children [64].

#### Goals of examination for ARVC

**Fig. 2:**
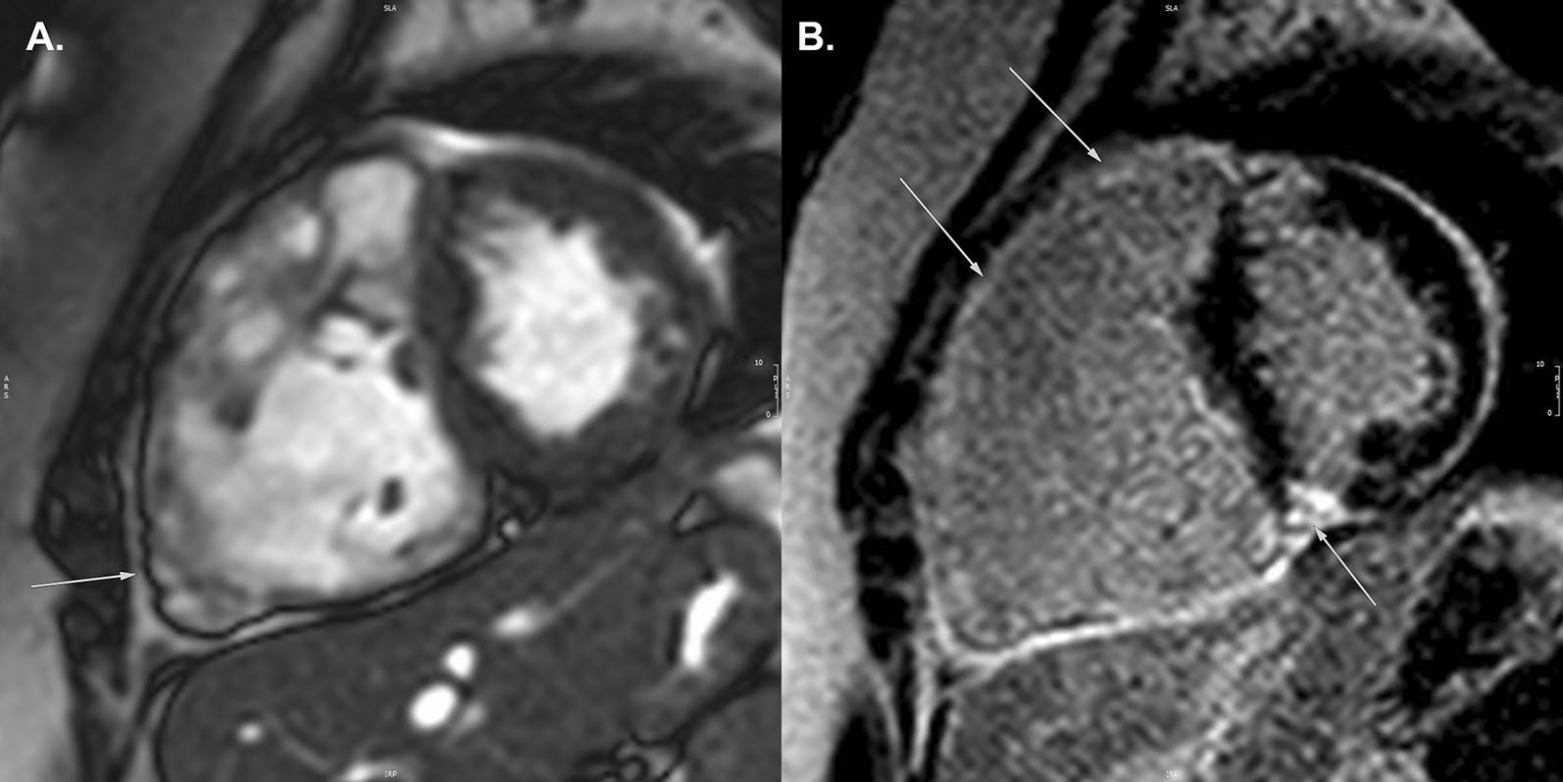
Arrhythmogenic right ventricular cardiomyopathy. **A** Systolic frame of a cine balanced steady state free precession (bSSFP) mid- ventricular short axis slice, showing severe right ventricular (RV) dilatation and small areas of infolding along the anterior wall (white arrow), or the so-called “accordion sign”. **B** Late gadolinium enhancement (LGE) image in the short axis plane, showing diffuse enhancement of the RV wall and dense enhancement in the area of the inferior septal insertion of the right ventricle

The goal of the CMR examination for suspected ARVC is to assess RV size, systolic function, and regional function (Table 3) (Fig. 2A) [65, 66]. Assessment should include a discussion of (1) abnormal wall thinning, (2) RV outflow tract dilation, (3) RV enlargement, and (4) RV global function and regional wall motion abnormalities. Fatty infiltration by CMR is not currently an ARVC Task Force criterion for diagnosis, and can be omitted from a typical protocol, shortening scan time considerably.

**Table 3:** Arrhythmogenic right ventricular cardiomyopathy

Sequence	Imaging plane	Indication
Standard imaging		
Cine bSSFP	Short-axis stack	LV volumes, mass, and EF RV volumes and EF
	LV long-axis views	Regional wall motion abnormalities
	Axial stack	RV regional wall motion abnormality and RV aneurysm RV volumes and EF
	2 Chamber RV view RVOT sagittal view	RV regional wall motion abnormality and RV aneurysm
Additional case-specific or comprehensive imaging		
T1-weighted imaging with and without fat saturation	Axial stack covering RV Short-axis stack	Fatty infiltration, although this is not part of the ARVC Task Force criteria
Late gadolinium enhancement	Short-axis stack LV long-axis views RV-specific views	Focal fibrosis, but also not part of diagnostic criteria

*ARVC* arrhythmogenic right ventricular cardiomyopathy, *EF* ejection fraction, *LV* left ventricular, *RV* right ventricular, *bSSFP* balanced steady state free precession

Myocardial LGE is also not part of the revised ARVC Task Force criteria, because detection in the thin RV wall can be challenging. In addition, LGE has been shown to have limited value in ARVC diagnosis in children [64]. Nonetheless, some have advocated assessment for areas of LGE (Fig. 2B), which have been shown to correlate with histological findings of myocardial fibrosis and inducible ventricular arrhythmias by electrical stimulation. There is also a strong association between areas of LGE and RV dysfunction in adults [65, 67, 68].

Comprehensive assessment of the RV requires multiple imaging planes, with axial cine images best for RV free wall evaluation, short-axis views for evaluation of the inferior wall and angle of the RV, and RV outflow tract (RVOT) views for the inferior wall. Regional wall motion abnormalities should be confirmed on images obtained in more than one imaging plane [69]. Subtle aneurysms of the RV wall (i.e., small focal bulges in the wall of the RV that persist in systole and diastole) or an “accordion sign” (i.e., focal crinkling of the RVOT or the sub tricuspid region of the RV free wall that becomes more prominent during systole) can be seen in patients who later go on to develop more phenotypic evidence for ARVC [70]. Regional RV wall motion abnormalities alone—in the absence of RV dilation or reduced RV systolic function—should not be taken as criteria for establishing the diagnosis of ARVC [69].

Careful scrutiny of the LV for regional wall motion abnormalities, LV dilation, global dysfunction, and possibly LGE as well (Fig. 2B), should be part of the ARVC CMR study. LV involvement has been considered a late-stage manifestation of the progressive disease [68, 71] but may be underdiagnosed [72], although these data are all in adult populations.

#### Limitations and pitfalls

There is a high potential for misdiagnosis of ARVC even with advanced CMR techniques, in part due to a low true positive prevalence in the referral population and difficulty in recognizing abnormal findings versus normal variants that can mimic ARVC [69]. Practitioners should seek secondary expert opinions for suspected controversial ARVC cases.

#### Newer techniques for ARVC

Myocardial deformation imaging to aid in the assessment of RV function is a promising modality for ARVC. With use of tagged CMR images, LV myocardial strain can be assessed, but application to the RV is limited given the very thin wall of the RV (2–3 mm) and the 6–10 mm spacing between tags [71]. However, FT has gained acceptance, replacing CMR tagging as a viable modality for measuring myocardial strain and has been applied in this population to both the LV [73] in children and the RV [74] in adults.

### Hypertrophic cardiomyopathy

Hypertrophic cardiomyopathy (HCM) is characterized by LV hypertrophy in the presence of a non-dilated ventricular chamber, not explained by fixed LV outflow obstruction or other cardiac disease. Most of the literature on CMR in HCM is focused on sarcomeric HCM (mutation in a gene encoding a sarcomeric protein), although there are also forms of HCM related to non-sarcomeric mutations, genetic syndromes or storage diseases. Patients may be genotype positive for HCM, but not yet express the hypertrophic phenotype. The pathophysiology of HCM is complex and may include the presence or absence of dynamic LV outflow tract (LVOT) obstruction, diastolic dysfunction, mitral regurgitation, myocardial ischemia, and arrhythmias.

The use of CMR has become standard in the adult population to confirm the diagnosis of HCM and for risk stratification. A series of papers has established the presence of LGE as an important risk factor for adverse outcomes in adults with HCM [75–79], and smaller studies have begun to establish the importance of this risk factor in children [80, 81].

The differentiation of HCM from physiologic LV remodeling in young athletes can be of critical importance. In challenging cases, this differentiation can be aided by a period of deconditioning, after which the athlete’s heart may show hypertrophy regression while the pathologic changes in HCM do not typically regress. CMR is the ideal imaging modality to assess for changes with deconditioning, given excellent contrast between endocardium and blood pool [82] and better intra- and inter-observer variability than TTE for measurements of wall thickness in children [83]. The latter is particularly important for comparing wall thickness across multiple CMR studies for this indication.

#### Goals of examination for HCM

CMR is well suited for diagnosis and prognosis in HCM. The primary goal of the CMR exam for a patient with suspected HCM is to characterize the LV and RV myocardium (Table 4). CMR is not limited by poor acoustic windows and provides imaging of all myocardial segments. CMR can define the pattern and extent of hypertrophy, assess the LVOT and mitral valve apparatus, and define extent of LGE.

**Table 4:** Hypertrophic cardiomyopathy

Sequence	Imaging plane	Indication
Standard imaging		
Cine bSSFP	Short-axis stack	LV volumes, mass, and EF RV volumes, mass, and EF
	LV 2-chamber view LV 4-chamber view	
	LV 3-chamber view (LVOT view)	LVOT obstruction and presence of systolic anterior motion of the mitral valve chordae and leaflets
Late gadolinium enhancement	Short-axis stack LV long-axis views RV-specific views	Extent of LGE via summation of short axis slices for calculation of percent myocardium with LGE; 2- and 4-chamber images for correlative data to rule out artifact
Additional case-specific or comprehensive imaging		
T1-weighted imaging pre- and post-gadolinium	Short-axis stack LV long-axis views	Diffuse fibrosis Extracellular volume
Tagged images (SPAMM or C-SPAMM) or feature tracking	Short-axis, LV long-axis	Myocardial deformation

*C-SPAMM* Complementary spatial modulation of magnetization, *EF* ejection fraction, *LGE* late gadolinium enhancement, *LV* left ventricular, *RV* right ventricular, *SPAMM* spatial modulation of magnetization, *bSSFP* balanced steady state free precession

#### Limitations and pitfalls

There are potential limitations of CMR specific to the pediatric population with suspected HCM. The importance of LGE as a risk factor for sudden cardiac death is not well established compared to the adult literature. In addition, children with HCM can be high risk during anesthesia [84], and case selection must be judicious, with anesthesia performed only when absolutely necessary by pediatric anesthesiologists experienced with cardiac disease.

#### Newer techniques for HCM

Myocardial deformation imaging has been applied in limited published studies in the assessment of HCM. Decreased global strain and strain rate are predictive of detectable LGE in children [85]. A small retrospective CMR study of myocardial strain using FT showed an association between decreased global radial strain (GRS) and global longitudinal strain (GLS) in a pediatric HCM cohort with hypertrophy and adverse outcome (i.e., ventricular tachycardia, appropriate defibrillator shock, and death) [86]. More data are needed to prove the utility of deformation imaging for prognosis in the pediatric HCM population. Recent studies have investigated the use of native T1 mapping in HCM in a pediatric population, showing that native T1 times and ECV measurement are higher in HCM patients than in controls, and in hypertrophied segments in HCM patients compared to non-hypertrophied segments, suggesting that this technique could be of use to assess for myocardial fibrosis [87]. This finding has not yet been established in genotype-positive, phenotype-negative patients or as a predictor of outcome in children. Quantitative CMR perfusion techniques showed impaired perfusion with adenosine-induced hyperemia in HCM patients compared to controls. These studies were predominantly comprised of adults but included a small number of pediatric patients [88, 89].

### Myocarditis

In myocarditis, a viral infection is believed to affect the myocardium and trigger an abnormal immune response, together leading to extracellular edema, myocardial necrosis and subsequent fibrosis. There are a variety of clinical presentations ranging from subclinical cases to severe disease leading to acute heart failure (HF) with the need for mechanical support or heart transplantation. Given that symptoms (chest pain, new-onset HF, or arrhythmia) are nonspecific, there are no standardized clinical diagnostic criteria. CMR imaging (Table 5) can contribute important information that together with other data (e.g., clinical history, electrocardiogram (ECG), laboratory testing, or endomyocardial biopsy (EMB)) may allow one to establish the diagnosis of myocarditis and monitor the course of the disease.

**Table 5:** Myocarditis

Sequence	Imaging plane	Indication
Standard imaging		
Cine bSSFP	Short-axis stack	LV volumes, mass, and EF RV volumes, mass, and EF Regional wall motion abnormalities
	LV long-axis views	
T2 edema-weighted imaging (e.g., STIR)	Short-axis stack LV 2-chamber view LV 4-chamber view	Focal myocardial edema Pericardial effusion
T1 mapping (native)	Short-axis stack	Focal and diffuse myocardial edema, but also detects hyperemia or fibrosis
T2 mapping (pre-contrast)	Short-axis stack	Myocardial edema
≥ 15 min post-contrast T1 mapping	Mid-cavity, apical and basal short-axis views	Extracellular volume
Late gadolinium enhancement	Short-axis stack LV long-axis views RV-specific views	Myocardial or pericardial hyperenhancement

*EF* ejection fraction, *LGE* late gadolinium enhancement, *LV* left ventricular, *RV* right ventricular, *bSSFP* balanced steady state free precession, *STIR* short tau inversion recovery

#### Goals of examination

The primary goals of CMR in myocarditis include quantification of biventricular volumes and function [90], and myocardial characterization by assessing edema, necrosis, or fibrosis [91, 92] (Table 5). The updated 2018 Lake Louise Criteria [93] define a study as positive if both a T1-based abnormality (by native T1 mapping, ECV, or LGE) and T2-based abnormality (by T2 mapping or T2-weighted imaging) are present. While those criteria were based primarily on adult data, the ability of T1 and T2 parametric mapping to diagnose myocarditis in a pediatric population has been confirmed [94]. Exclusion of other causes of new-onset HF, chest pain, or arrhythmia are essential. While in adults the primary alternatives would be coronary artery disease or Takotsubo cardiomyopathy, pediatric patients should be carefully examined for the presence of CHD, such as anomalous origin of the left main coronary artery from the pulmonary artery.

The most challenging problem remains the differentiation between myocarditis and non-inflammatory (hereditary) DCM. Abnormalities on T2-weighted imaging or T2 mapping show evidence of myocardial edema and indicate acute disease more suggestive of myocarditis. Abnormalities of T1-weighted imaging provide evidence of non-ischemic myocardial injury. T1 mapping [95–97] should be incorporated into the protocol if available. The diagnosis of myocarditis is also supported by the finding of LGE in the typical pattern of subepicardial striae located in the infero-lateral LV wall. Adult data suggest that the presence of LGE is a risk factor for adverse events in patients with myocarditis [98], and a small retrospective study in a pediatric population showed the same finding [99].

A minority of patients present with extensive or circular hyperenhancement, indicating a severe global inflammatory process. In these cases, giant cell myocarditis and non-viral origins such as sarcoid disease should be considered, although rare in children. However, there is a significant proportion of patients presenting with advanced DCM phenotype (LV dilatation and severely impaired systolic function) without signs of myocardial injury on CMR but with marked inflammatory findings on EMB. This may be particularly seen in young children and infants, possibly indicating a failure of the immature immune system in these patients.

#### Limitations and pitfalls

Modifications in CMR technique are often required to adjust for small body size and high heart rate in young children, as discussed in the introductory portion of this document. Imaging without breath holding is particularly challenging for myocarditis. While cine imaging usually yields good image quality during free breathing (using multiple signal averages), black-blood techniques, such as short tau inversion recovery (STIR), are very sensitive to breathing artifacts and might be better replaced by T2-prepared gradient echo techniques in cases of limited compliance of the patient. Parametric imaging is very limited without breath holding, unless automated motion correction is available. While there is hope that CMR will provide valuable prognostic information for individual patients, there remain little outcomes-based data in children with suspected or confirmed myocarditis.

#### Newer techniques

While T1 and T2 mapping are discussed as newer techniques in other sections of this document, the role of these techniques in the diagnosis of myocarditis is well accepted [93, 94]. The 2018 Lake Louise criteria can be fulfilled with traditional T2-weighted sequences and LGE. However, based on current data and criteria, the use of T1 and T2 mapping is recommended as part of a comprehensive study for assessment of possible myocarditis.

### Kawasaki disease and systemic vasculitis

Kawasaki disease (KD) is an autoimmune systemic disease involving coronary and peripheral vessels, pericardium, and all layers of the myocardium. The use of intravenous gamma immunoglobulin (IVIG) early in the course of disease has modified the phenotypic expression of KD. However, coronary artery aneurysms (CAA), myocardial inflammation, and myocardial infarction continue to present as life-threatening complications of this disease. KD predominantly occurs in young children and produces CAA in 15–25% of untreated cases [100], which has been reduced to ~ 4% with early IVIG treatment [101]. Myocardial ischemia may be caused by ruptured or thrombosed CAA or by stenotic lesions on either end of the CAA. Serial evaluation of the distribution and size of CAA and screening for coronary artery stenosis is necessary for risk stratification and therapeutic management [100–102].

Systemic vasculitides in children are uncommon diseases linked by the presence of blood-vessel inflammation. The systemic vasculitides may be categorized according to the predominant size of the blood vessels involved: small, medium, or large [103]. CMR has the potential to play a role in initial assessment and follow-up of patients with KD, a medium-vessel disease, and other systemic vasculitides involving medium or large vessels, such as childhood polyarteritis nodosa and Takayasu arteritis, respectively [103, 104]. Currently, there is no gold standard available to confirm vessel wall thickness in children, but CMR has the potential to fill this gap.

#### Goals of examination

**Fig. 3:**
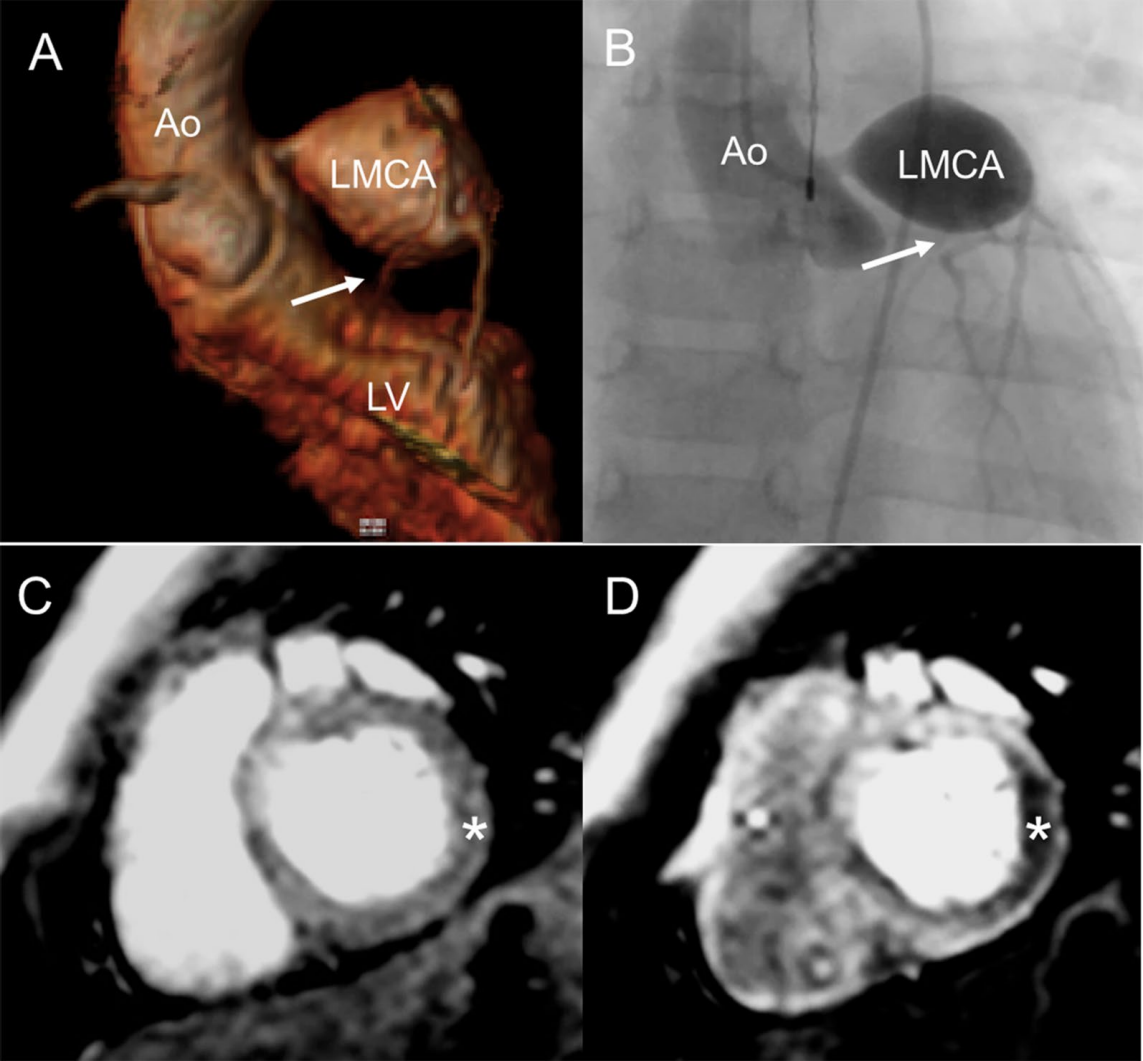
Coronary artery aneurysm and perfusion defect in Kawasaki disease. A volume rendered reformat of a 3D whole heart bSSFP sequence with electrocardiogram (ECG) triggering, prospective respiratory navigator correction, flow insensitive T2-prepulse and a spectrally selective fat-saturation pulse was used to visualize the coronary artery lumen in a 3 year old patient with Kawasaki disease. A giant coronary artery aneurysm of the left main coronary artery (LMCA) was detected (**A**). The findings were confirmed by cardiac catheterization (**B**). Myocardial perfusion (balanced *kt* perfusion sequence, acceleration factor 5) was normal at rest (star in **C**). The suspicion of a left circumflex stenosis (arrows in A and B) was confirmed with adenosine stress perfusion (star in **D**)

CMR is the ideal imaging modality to visualize the coronary artery system in relation to the surrounding soft tissues and vasculature. The clinical utility of coronary cardiovascular magnetic resonance angiography (CCMRA) in KD has been shown in several feasibility studies. CMR is highly useful for demonstrating coronary artery aneurysms and other coronary pathology (Fig. 3) [105–107]. Combined with the established ability of CCMRA to visualize aneurysms in the coronary artery system, CMR may also delineate thrombi within the aneurysms with black blood techniques [108]. CMR, combined with CCMRA, can offer a detailed evaluation of the coronary and other blood vessel lumens, vessel walls, myocardial perfusion, ventricular function, myocardial inflammation, and fibrosis (Table 6).

**Table 6:** Kawasaki disease and systemic vasculitis

Sequence	Imaging plane	Indication
Standard imaging		
2D Cine bSSFP	Short-axis stack	LV volumes, mass, and EF RV volumes, mass, and EF Regional wall motion
	LV long axis views	
3D bSSFP respiratory-gated and ECG-triggered whole heart sequence^a^	3D Volume	Coronary artery assessment for aneurysm formation
Non-ECG gated 3D magnetic resonance contrast angiography	3D Volume	Extracardiac vascular lumen imaging for detection of aneurysm formation
Late gadolinium enhancement	Short-axis stack LV long-axis views RV-specific views	Myocardial scarring
Additional case-specific or comprehensive imaging		
Navigator gated 3D LGE [110, 111]		Coronary or large vessel wall enhancement in patients with systemic arterial inflammation
T1 mapping	Short-axis stack	Acute myocardial inflammation (edema) or fibrosis
T2 signal intensity ratio OR T2 mapping	Short-axis stack	Acute myocardial inflammation (edema)
Rest and stress myocardial perfusion	Short-axis stack	Suspected coronary stenosis including microvascular disease of the myocardium
Phase contrast flow	As indicated	KD-related decreased ventricular output or valve insufficiency

*3D* three dimensional, *ECG* electrocardiogram, *EF* ejection fraction, *KD* kawasaki disease, *LV* left ventricular, *RV* right ventricular, *bSSFP* balanced steady state free precession

^a^Imaging can be performed during systole or diastole according to patient age and heart rate

In Takayasu arteritis, contrast-enhanced CMR using black blood techniques (electrocardiogram (ECG)-gated spin-echo sequences with presaturation pulses for magnetization preparation) are capable of early diagnosis of inflammatory changes in the arterial wall, even in segments without significant wall thickness [109].

#### Limitations and pitfalls

Imaging coronary arteries in children can be challenging due to a high heart rate and small vessel size, as well as respiratory motion artifacts. Imaging during systole may be beneficial particularly in young patients with high heart rates [112]. There is only limited experience for assessment of coronary artery stenosis and myocardial perfusion in pediatric patients [113, 114], and future multicenter studies are necessary to prove clinical value using these techniques.

#### Newer techniques

Further improvement of coronary artery lumen imaging can be achieved by acquiring both cardiac rest periods during systole and diastole [115] and with the use of new navigator techniques to correct for respiratory motion [116–120]. In addition to lumen imaging, vessel wall thickness can be assessed with CCMRA, which allows further assessment of the coronary artery system for risk stratification and monitoring of treatment. Several studies show increased vessel wall thickness compared to healthy subjects [105, 121, 122]. Coronary artery stenoses have been shown to develop in the area of CAA as a long term consequence of KD [101, 123]; imaging the vessel wall may help to quantify future risk for coronary artery stenosis not only based on CAA size but also on diseased vessel wall thickness [109]. Additional techniques, which may provide insight in disease development and outcome, include T1 and T2 mapping and coronary and aortic vessel wall imaging [101, 121, 124].

### Coronavirus disease-2019 (COVID-19)

Myocardial inflammation, dysfunction, conduction and rhythm disorders are prominent features of severe acute respiratory syndrome-coronavirus-2 (SARS-CoV-2). Although early in the course of the COVID-19 pandemic myocardial involvement was thought to almost exclusively involve adults, evidence of myocarditis and its complications soon emerged in the pediatric population [125]**.** In those with less severe disease, there is concern for children and teenagers with prolonged symptoms following COVID-19 infection (so-called “long-COVID”). Symptoms can include palpitations, chest pain and dyspnea on exertion [126]. Published data on management and outcomes of these findings in the pediatric population are scant, including mostly case reports and a small case series [127].

Multisystem inflammatory syndrome in children (MIS-C) is a novel syndrome described in children following infection with SARS-CoV-2, as the consequence of a strong systemic inflammatory reaction. The clinical presentation has been compared to KD and other inflammatory syndromes, though the overlap between MIS-C and KD is incomplete. Presentation includes fever in children who have been recently infected with SARS-CoV-2, accompanied by symptoms similar to KD such as rash, edema of hands and feet, oral mucosal changes, conjunctivitis, lymphadenopathy and neurologic symptoms [128]. Cardiac involvement is frequent and can include myocardial inflammation and dysfunction as well as coronary artery dilation. CMR has been described in the acute setting with high rates of myocardial edema and global dysfunction [129]. Hypotension or shock can be a prominent feature of this illness. Treatment of the patient with severe disease with IVIG and steroids has proven to be effective in the majority of cases [130].

#### Goals of examination

The goals of CMR for indications related to COVID-19 infection and MIS-C are similar to myocarditis, including assessment of myocardial function and for evidence of edema, inflammation and LGE. Use of CMR for the presentation of acute myocarditis during COVID-19 infection is rarely indicated in children. Additionally, CMR is not currently recommended as a primary screening tool for pediatric patients with long-COVID, but may be useful if abnormalities are uncovered by other cardiac testing. In the pediatric population, the use of CMR in this disease has been focused on the convalescing adolescent who wishes to return to athletic competition after recovery; this indication is driven by concern that even in those who were asymptomatic to their COVID-19 infection, there could be occult myocardial involvement that places them at risk of sudden events with exertion. A retrospective study of 1597 college athletes who had CMR following COVID-19 diagnosis included 37 (2.3%) positive studies [131], based predominantly on LGE and elevated T2 findings. Of the 37 athletes with CMR findings of myocarditis, 31 met modified Lake Louise Criteria for myocarditis. Further, compared with CMR, screening strategies based on symptoms and abnormal laboratory findings failed to identify more than 50% of cases. A prospective study of 3018 college athletes who tested positive for COVID-19 [132] included a primary screening CMR in 198 subjects, with positive findings in 6 (3%); an additional 119 of the other 2820 subjects were referred for CMR due to other abnormal cardiac testing and 15 of those 119 (13%) had positive CMR findings. The authors noted the low prevalence of cardiac findings overall and that CMR was more useful in patients who had screened positive otherwise for cardiac involvement. Importantly, there were no cardiac adverse events in these 3018 subjects during the period of surveillance.

Published recommendations for cardiac testing prior to return to play are available from the Sports and Exercise Cardiology Section of the American College of Cardiology [133]. The recommendation for the use of CMR for high school and college athletes prior to return to play is restricted to those with positive findings from TTE, ECG, or serum troponins, or with new cardiovascular symptoms developing during a slow resumption of activity if other testing has already been performed. A consensus has developed that CMR is not indicated for primary screening of asymptomatic patients following COVID-19 infection.

The role of CMR for children with MIS-C is not yet precisely defined. In most cases, there is not a clear indication for CMR in the acute setting; cardiac involvement is typically clinically defined, and dysfunction well seen by TTE. While the rate of myocardial inflammation seen by CMR is high, treatment of this syndrome is based on anti-inflammatory agents and acute CMR findings do not typically impact on management, except in the relatively rare case of diagnostic uncertainty. However, current recommendations [128] include CMR 2–6 months post-acute illness in patients who had LVEF < 50% at presentation or persistent LV dysfunction. This is to assess function as well as for persistent evidence of inflammation or fibrosis, including LGE. The same document recommends cardiac CT separately for investigation of suspected distal coronary artery aneurysms.

The protocol for CMR studies of patients to assess for myocardial involvement after COVID-19 infection or following MIS-C is the same as myocarditis (Table 5). Coronary artery imaging for MIS-C patients should be considered.

#### Limitations and pitfalls

No studies to date have linked positive CMR findings in recovering pediatric COVID-19 patients to clinically important outcomes. The question remains of whether positive CMR findings of inflammation or LGE in asymptomatic or convalescing patients are evidence of significant clinical disease.

### Cardiac tumors

**Fig. 4:**
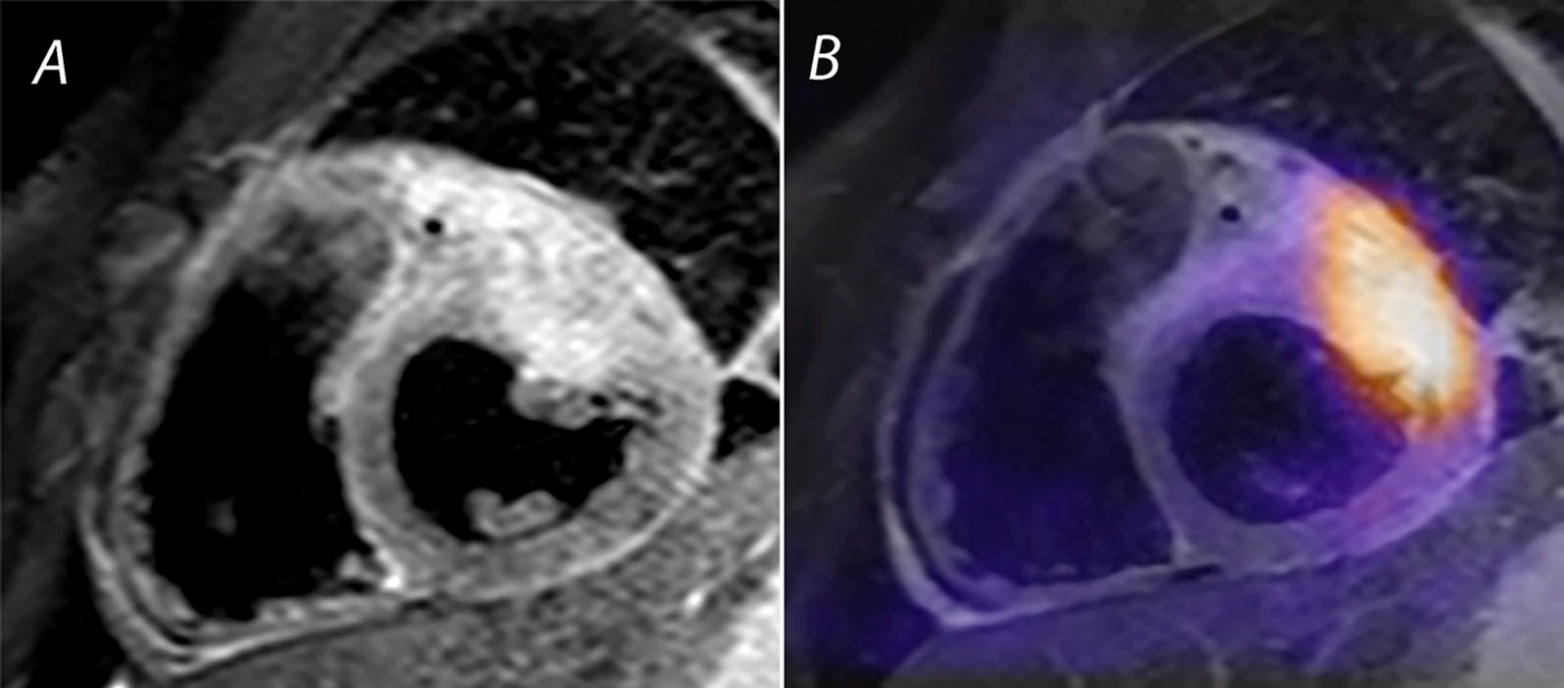
Rhabdomyosarcoma: primary malignancy of the heart. **A** Double inversion recovery turbo spin echo image with T2 weighting and a fat saturation pulse, showing a rhabdomyosarcoma in the anterior wall of the left ventricle (LV). Note the heterogeneity of signal and lack of distinct tissue boundaries. **B** Positron emission tomography (PET)-CMR image of the same tumor showing avid uptake of 5-fluorodeoxyglucose in the malignant lesion

Cardiac tumors are a proliferation of tissue arising from various cellular precursors, including muscle (rhabdomyoma), fibrous (fibroma), vascular (hemangioma), fat (lipoma), nervous (paraganglioma), and ectopic (teratoma) tissues. Most cardiac tumors in children are histologically benign; however, primary and secondary malignant cardiac tumors also occur [134] (Fig. 4). Cardiac tumors in children are rare, with a prevalence of up to 0.08% in autopsy studies and 0.32% in series that utilized TTE [135]. While TTE is the primary imaging modality for detecting cardiac tumors, CMR provides better imaging of tumor size and location, anatomic relation to adjacent cardiac and mediastinal structures, and tumor signal characteristics.

#### Goals of examination

A comprehensive CMR examination protocol (Table 7) aims to determine tumor location, size, hemodynamic consequences (e.g., obstruction of blood flow, ventricular dysfunction), mobility, anatomic relations to neighboring cardiovascular and mediastinal structures, and signal characteristics. Table 8 summarizes key CMR features suggestive of the likely tumor type [136].

**Table 7:** Cardiac tumors

Sequence	imaging plane	Indication
Standard imaging		
Cine bSSFP	Axial and oblique planes across the tumor	Tumor size and location Assessment for potential interference with blood flow or valve function
	Short-axis stack LVOT and RVOT long-axis	Assessment for potential interference with blood flow, valve function, or ventricular function
T1-weighted spin echo with and without fat suppression	Across the tumor and uninvolved myocardium	Tumor characterization
T2-weighted spin echo with fat suppression	Same plane and coverage as T1-weighted sequence	Tumor characterization
First-pass perfusion	Short-axis 4-chamber	Myocardial and tumor perfusion
Late gadolinium enhancement	Across the tumor and uninvolved myocardium	Fibrosis
Additional late gadolinium enhancement imaging 20–30 min post-contrast	As indicated across the tumor and uninvolved myocardium	Strong hyperenhancement may be present in certain cases of large fibromas
Post-contrast late gadolinium enhancement with long inversion time (600 ms)	As indicated across the tumor and uninvolved myocardium	Suspicion of thrombus
Additional case-specific or comprehensive imaging		
Coronary artery imaging		Coronary artery relationship to tumor
Velocity encoded phase contrast flow		Tumor-related obstruction to blood flow and valvular regurgitation
Magnetic resonance contrast angiography		Extracardiac vascular anatomy

^a^Post-contrast T1- or T2-TSE sequences are not recommended since they do not provide additional diagnostic information.

*LVOT* left ventricular outflow tract, *RVOT* right ventricular outflow tract, *bSSFP* balanced steady state free precession

**Table 8:** CMR features of cardiac tumors and masses in children^a^

Tumor type	Location	bSSFP	T1	T1 + Fat sat	T2	FPP	LGE	Other
Fibroma	**Intramyocardial, ventricular septum or free wall**	–	±	±	±	**No**	** + + ** **(well-defined border ± dark core)**	Can be in an atypical location (e.g., atria)
Rhabdomyoma	Intramyocardial or intracavitary, attached to myocardium, often multiple tumors	±	±	±	+	**No**	––	
Malignant	Infiltrative^b^		±		±	Variable	± (if + then heterogenous appearance)	**Known malignancy,** pericardial effusion
Vascular^c^	Variable	±	–	–	+ (variable)	**Strong**	+ (variable and heterogenous)	Consider malignant tumor
Thrombus	**Mural or intraluminal**	–	–	–	–	**No**	––	LGE sequence, long inversion time
Myxoma	Typically left atrium but can be in any chamber	±	±	±	+	No	** ± **	**Irregular, pedunculated, mobile**
Fibroelastoma	Pedunculated, mobile endocardial or valvular mass	–	–	–	–	No		
Pleuropericardial cyst	Right cardiophrenic angle	** + + **	–	–	** + + **	No	–	Smooth-walled and well-defined
Purkinje cell tumor	Ventricular myocardium		** + + **	––	–	No		**Ventricular arrhythmia**
Teratoma	Intrapericardial (usually compressing SVC and/or right atrium)	±				No		Multilocular bosselated mass with solid and cystic areas
Lipoma	Any chamber	–	** + + **	––	±	No	–	

Key: – denotes iso- or hypointense, ± denotes variable intensity, + denotes hyperintense, +  + denotes strongly hyperintense

Bolded fields signify either strongly supportive of or necessary for diagnosis

*FPP* first pass perfusion, *LGE* late gadolinium enhancement, *SVC* superior vena cava

^a^Modified from Beroukhim et al.[136]

^b^Anatomic features of an infitrative tumor include (1) crossing an annular or tissue plane within the heart; (2) involving both cardiac and extracardiac structures; or (3) appearance of linear growth through a large vessel such as the superior or inferior vena cava

^c^Vascular refers to tumors with abundant vascular supply, including hemangioma, malignant vascular tumors, and paraganglioma

#### Limitations and pitfalls

Although exceedingly rare in the pediatric age group, malignant cardiac tumors (primary or metastatic) can occur [134, 137–139]. In the absence of a known extracardiac malignancy, the distinction between benign and malignant cardiac tumor can be challenging. Features that should prompt consideration of malignancy include (1) absence of clear demarcation or a tissue plane between the tumor and adjacent myocardium or other cardiac structures; (2) involvement of both cardiac and noncardiac structures; (3) extension through large blood vessels such as the inferior vena cava; and (4) pericardial effusion associated with an atypical mass. Another limitation is CMR’s inability to distinguish between specific types of highly vascular tumors such as hemangioma, vascular tumors with malignant potential (e.g., angiosarcoma), certain vascular malformations [140], and some neuroendocrine tumors (e.g., paraganglioma) [137]. Because of the possibility of malignant potential, consideration should be given to histologic examination of any tumor with CMR evidence of a strong vascular supply on first-pass perfusion imaging.

### Pericardial disease

The indications for CMR in pediatric pericardial disease include suspicion of constrictive pericarditis, differentiation of constrictive pericarditis from restrictive cardiomyopathy, failure to respond to anti-inflammatory therapy in infective pericarditis, and evaluation for inflammation in effusive-constrictive pericarditis [141–143]. There are multiple strengths of CMR for imaging in pericardial disease, including a combination of high-quality tissue characterization with additional functional information, unique contrast resolution between fat, fibrous, and hemorrhagic components of the pericardial wall and a role in predicting pericardial inflammation reversibility [144]. These data are largely drawn from an adult population.

For the diagnoses of pericardial effusion, constrictive pericarditis, and acute pericarditis, the goals of the examination are provided below. A combined standard and case-specific CMR imaging protocol is provided in Table 9.

**Table 9:** Pericardial disease combined standard and case-specific imaging

Sequence	Imaging plane	Indication
Spin echo CMR or cine bSSFP	Axial	Pericardial thickness, fluid extent, localization
Cine bSSFP	Short-axis views	Ventricular and atrial size and shape, ventricular function
	LV long-axis views RV-specific views	
Cine CMR with tagging	Short-axis stack LV 4-chamber view	Screen for fusion of pericardium and myocardium in constriction
Real-time free-breathing bSSFP	Short axis	Ventricular coupling: ventricular septal shape motion pattern with respiration in constriction
T1- or T2-weighted with post Gadolinium spin echo or cine SSFP	Axial	Pericardial layer or fluid characterization
Late gadolinium enhancement	Axial	Distinguish fat vs. fibrosis vs. inflammation
Velocity encoded phase contrast flow	4-chamber view	Abnormal atrioventricular valve filling in constrictive pericarditis

*CMR* cardiac magnetic resonance, *LV* left ventricular, *RV* right ventricular, *bSSFP* balanced steady state free precession

### Pericardial effusion

#### Goals of examination

CMR can localize and quantify the amount of pericardial fluid, differentiate pericardial thickening from effusion, and characterize fluid by signal intensity. CMR is a superior technique to TTE for detecting distribution and the amount of fluid accumulation, especially loculated effusions. CMR, as a complimentary modality, may also aid in the detection of abnormal filling patterns and early signs of tamponade physiology in patients with a pericardial effusion [145], although it is not a good option for a patient with signs of tamponade physiology and any hemodynamic instability.

Common practice guidelines exist to categorize pericardial effusion by size in adults. However, similar guidance is not widely available for size of a pericardial effusion relative to body size in pediatrics. Describing the size and location of the fluid qualitatively is clinically useful [145]. CMR can also characterize the effusion. Transudate is characterized by low signal intensity on T1 and high signal intensity on T2-weighted spin echo; the inverse is true for exudates. Signal intensity differs for hemorrhagic pericardial effusion depending on the duration of the disease.

### Constrictive pericarditis

Constrictive pericarditis occurs when a thickened fibrotic pericardium impedes normal diastolic filling. This usually involves the parietal pericardium, although it can involve the visceral pericardium, as in constrictive-effusive pericarditis. Acute and subacute forms of pericarditis may deposit fibrin, which may, in turn, evoke a pericardial effusion. This often leads to pericardial organization, chronic fibrotic scarring, calcification, and restricted cardiac filling.

The clinical symptoms and classic hemodynamic findings can be explained by early rapid diastolic filling and elevation and equalization of the diastolic pressures in all of the cardiac chambers, restricting late diastolic filling and leading to venous engorgement. These pathophysiologic features are associated with decreased cardiac output secondary to a confining pericardium. CMR can play an important role in making the diagnosis when clinical and echocardiographic evaluation is indeterminate.

#### Goals of examination for constrictive pericarditis

CMR is useful for direct anatomic imaging of the pericardium, as well as assessment of physiologic changes that are related to pericardial constriction. The following findings are helpful for making a diagnosis of constrictive pericarditis in children:

1. Pericardial thickness: Criteria to assess pericardial thickness by CMR based on adult data are
  Pericardial thickness 2 mm or less: normal
  Pericardial thickness greater than 4 mm: suggestive of pericardial constriction in patients with appropriate clinical presentation
  Pericardial thickness greater than 5–6 mm: high specificity for constriction.
2. The thickened fibrotic or calcified pericardium has a low signal not only on T1- and T2-weighted spin-echo CMR but also on cine imaging. In end stages, there may not be LGE.
3. Constrictive pericarditis is typically characterized by accentuated respiratory-related variation in cardiac filling (i.e., enhanced RV filling with inspiration). *Real time* cine CMR can assess the effects of respiration on filling. In constrictive pericarditis, there is increased ventricular coupling characterized by septal flattening or inversion on early diastolic ventricular filling (“septal bounce”), which is strongly influenced by respiration. Giorgi and associates, using free-breathing cine CMR, reported early diastolic septal flattening in the majority of cases of constrictive pericarditis in adults [146], which can likely be extrapolated to children.
4. Dynamic CMR with tagging can also evaluate pericardial mobility. Rigid pericardium shows no or at most a limited displacement during the cardiac cycle.
5. LGE: Zurick and Klein reported that the presence of pericardial LGE in a pediatric patient was associated with histological findings of pericardial inflammation [147]. LGE may be useful in differentiating between ongoing pericardial inflammation and pericardial fibrosis based on adult data, thus allowing for tailored treatment options in patients with constrictive pericarditis [144, 148].
6. Velocity encoded phase contrast flow imaging can show changes in mitral or tricuspid valve inflow, similar to spectral Doppler on echocardiography, which can suggest a restrictive or constrictive filling pattern. However, data showing correlation to invasively measured pressures are lacking. In addition, standard sequences do not allow for measuring respiratory variation of inflow, which is a significant limitation of this methodology by CMR.

### Acute pericarditis

Prior descriptions of the use of CMR in pediatric patients with pericarditis have been limited to case reports [147, 149]. Prior cardiac surgery had been noted to be a significant risk factor for pericarditis in prior studies, as has complicated idiopathic/viral pericarditis.

#### Goals of examination for acute pericarditis

**Fig. 5:**
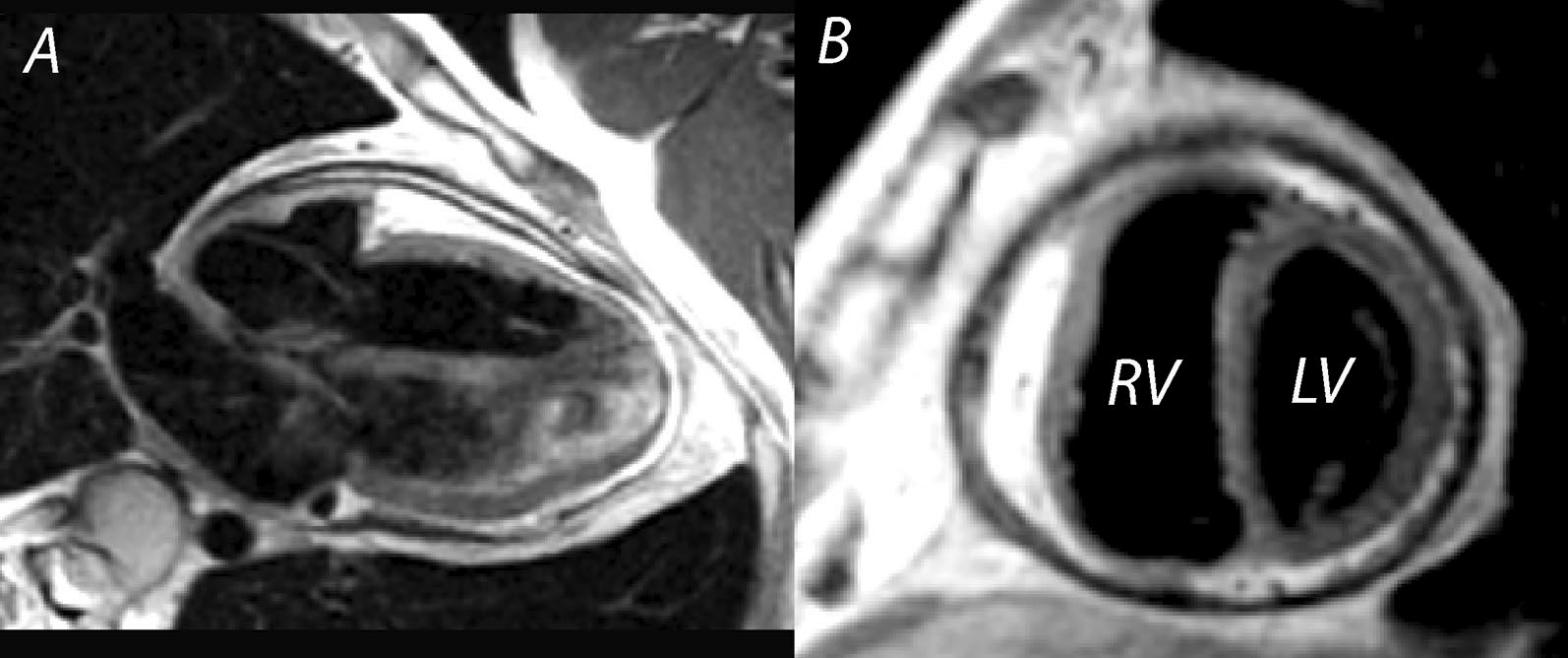
Pericardial disease. Horizontal long axis (**A**) and short axis (**B**) double inversion recovery turbo spin echo images demonstrating thickening of the pericardium and pericardial effusion

In acute pericarditis, the goals of the examination are to characterize both the pericardium and the pericardial effusion. CMR demonstrates enhancement of thickened pericardium on T1-weighted images or LGE, with a sensitivity of 94% to 100% in detecting pericardial inflammation in an adult population (Fig. 5) [150]. Increased signal in pericardial tissue on T2-weighted STIR images correlates with pathologic findings of edema, neovascularization, or granulation tissue. Among patients with acute pericarditis, myocardial involvement may also be an indication for CMR [151]. CMR is useful in the evaluation of pediatric patients with elevated serum troponin and chest pain that can be seen with coronary anomalies, coronary vasospasm, and arrhythmia in addition to myopericarditis or myocarditis [152].

Recurrent pericarditis is rare in children and can have an unpredictable course [153]. Pericardial enhancement in CMR can provide supportive evidence of recurrent pericarditis and also may be useful in ruling out pericardial inflammation.

#### Limitations and pitfalls of pericardial CMR

CMR imaging has poor sensitivity to the presence of calcium in the pericardium. In addition, in children who may need anesthesia for their CMR study, the use of positive pressure ventilation leads to difficulty interpreting septal motion with real time free-breathing bSSFP imaging. Use and interpretation of this sequence with positive pressure ventilation is not addressed in the literature. There is generally a lack of pediatric-specific literature on this topic.

### Pulmonary hypertension

Pulmonary hypertension (PH) is a disease of both the pediatric and adult population, often with a severe prognosis. Compared to adults, PH in children is less likely to be primary or thromboembolic in origin, while it is more likely to be related to CHD or prematurity with lung disease [154]. While initial presentation may vary, eventual morbidity and mortality from this disease is typically related to right heart failure. Invasive testing for assessment of pulmonary artery pressure and pulmonary vascular resistance has long played a central role in the initial diagnosis and longitudinal evaluation of this disease. The use of CMR in PH has grown rapidly and has been included as a Class I recommendation for initial diagnostic workup of PH in children who do not require sedation or anesthesia [155].

#### Goals of examination

Examination of the RV and the pulmonary arteries is crucial for the clinical care of pediatric patients with PH. CMR offers the ability to accurately measure the size and systolic function of the RV, with RVEF shown to be a predictor of survival in this pediatric population [156]. In addition, these clinical variables can be tracked over time, allowing the clinician to follow the effects of therapy as well as potentially offering prognostic information. Direct imaging of the pulmonary arteries can be helpful for initial diagnosis. In cases associated with CHD, such as tetralogy of Fallot with abnormal pulmonary vasculature, CMR can image areas in which direct intervention may be indicated. However, computed tomography (CT) angiography is thought to be the better test for chronic thromboembolic disease.

In all cases, measuring the size and function of both ventricles is important (Table 10). The pulmonary arteries should be imaged at least at the time of the first CMR scan, and depending on etiology, potentially on subsequent scans. Other examination goals include physiologic information such as pulmonary regurgitation volume or fraction and differential branch pulmonary arterial flow.

**Table 10:** Pulmonary hypertension

Sequence	Imaging plane	Indication
Standard imaging		
Cine bSSFP	Axial, vertical, and horizontal long axes of the RV	RV volumes, mass, and EF Assessment of pulmonary artery anatomy and pulsatility
	Short-axis stack	LV volumes, mass, and EF RV volumes, mass, and EF
Phase contrast flow	Ascending aorta, main pulmonary artery, and branch pulmonary arteries	Pulmonary regurgitation Differential pulmonary blood flow Qp:Qs measurement in context of a suspected shunt
Additional case-specific or comprehensive imaging		
Phase contrast flow	Pulmonary veins	Aortopulmonary collateral flow measurement
Magnetic resonance contrast angiography	3D volume	Indicated as part of first study Pulmonary artery anatomy Pulmonary vein anatomy
Late gadolinium enhancement	Short-axis stack LV long-axis views RV-specific views	Myocardial scarring
3D bSSFP imaging	3D volume	Intracardiac anatomy
4D flow imaging	Pulmonary arteries RV inflow	Assessment of pulmonary arterial pressures Wall shear stress RV diastolic function

*3D* three-dimensional, *4D* four-dimensional, *EF* ejection fraction, *LV* left ventricular, *RV* right ventricular, *bSSFP* balanced steady state free precession

The pulmonary-to-systemic flow ratio (Qp:Qs) should be reported in the case of either a left-to-right shunt as potential etiology of PH, or right-to-left shunting across the atrial septum associated with cyanosis in more advanced forms of the disease. Gadolinium-enhanced cardiovascular magnetic resonance angiography (CMRA) is indicated for a first study to assess the anatomy of the pulmonary arteries and veins and assess for any extracardiac sources of shunting, such as a ductus arteriosus or major aortopulmonary collateral artery. It may be indicated in follow up studies to assess progression of disease. Time-resolved CMRA may be helpful to qualitatively assess differential pulmonary perfusion, and may also have advantages for patients who cannot perform a long breath hold. 3D, gated, bright blood imaging such as 3D bSSFP is used particularly if intracardiac lesions are suspected. 3D gated imaging can also replace the gadolinium-enhanced CMRA if there is a contraindication to the use of contrast, but at the cost of lower SNR and inferior visualization of the pulmonary arterial and venous anatomy.

#### Limitations and pitfalls

Breath holding can be difficult for many patients with PH. Cine bSSFP data can be degraded by respiratory motion artifact, resulting in decreased accuracy and reproducibility of ventricular measurements. Free breathing techniques with multiple signal averaging can be used, though still with some degree of respiratory motion artifact. Real time imaging is another option, although this results in decreased temporal and spatial resolution.

Phase contrast flow mapping can be inaccurate in the context of a large, dilated main pulmonary artery with flow vortices. Measuring flow in the ascending aorta and the venae cavae can allow for checking internal consistency of data, in the absence of any shunting.

Patients with severe PH are high risk during anesthesia [157]. In younger patients with this disease who require anesthesia, CMR should be reserved for specific indications that will impact medical therapy or intervention. Scanning patients who require continuous pulmonary vasodilator infusion therapy requires coordination with pharmacy; changing to a CMR-compatible pump is necessary and it is critical to preplan, ideally with a local protocol, to avoid interruption of therapy when entering and exiting the scanner.

#### Newer techniques

Myocardial deformation, or strain imaging, is a promising technique for assessing regional ventricular function, and may also provide a means for earlier detection of dysfunction compared to RVEF [158]. This has been performed with tagged imaging, although there are limitations for the thin-walled RV relative to the space between tags [159]. Tissue tracking has also been applied to the RV [160]. This and related techniques have the advantage of using standard bSSFP images, so they do not require additional imaging while the patient is in the scanner. There is limited pediatric data regarding strain imaging in PH.

Tissue characterization with T1 mapping is also of interest in this disease [161]. LGE imaging can detect discrete areas of scarring or fibrosis, but cannot detect diffuse changes in the myocardium. T1 mapping techniques have been applied to both ventricles, but may be more reliable in the thicker LV due to inaccuracy related to partial volume effect of the RV myocardium with the blood pool. Challenges of T1 mapping in children are discussed in the introduction of this document.

Four-dimensional flow (4D flow) is an emerging technique in the assessment of patients with suspected PH [162]. Presence of abnormal vortices in the pulmonary arteries is a marker of elevated pulmonary arterial pressures. In addition, 4D flow can be used to assess pulmonary arterial wall shear stress and detect RV diastolic dysfunction in patients with PH. Future studies are needed to evaluate the role of 4D flow assessment by CMR in pediatric patients with PH.

### Heart transplantation

Heart transplantation is the final pathway for both failed CHD palliation and end-stage cardiomyopathy in pediatric patients [163]. CMR is a promising modality for providing unique information to clinicians caring for this patient population.

#### Goals of examination

The goals for noninvasive imaging in pediatric transplant patients include (1) accurate assessment of heart function; (2) myocardial characterization for rejection and transplant coronary allograft vasculopathy (CAV); (3) monitoring cardiac valve function; and (4) evaluation for sequela of failed palliation (Table 11). Transplant patients are referred to CMR when TTE is inadequate for function measurement, when trying to avoid catheter biopsy, or when the biopsy data and the clinical scenario are discordant. The possible avoidance of frequent serial biopsy is particularly compelling in pediatric patients, who may have greater need for sedation for biopsy and greater concern for additive use of ionizing radiation over a lifetime compared to adults. Pediatric heart transplants in patients who are status-post failed palliation for CHD are sometimes referred for CMR surveillance of systemic or pulmonary venous repair or pulmonary or systemic arterial repair, which were part of the palliation but not completely repaired or replaced at the time of the transplant.

**Table 11:** Heart transplantation standard imaging

Sequence	Imaging plane	Indication
Standard imaging		
Cine bSSFP	Short-axis stack LV long-axis views	LV volumes, mass, and EF RV volumes, mass, and EF Regional wall thickness and motion
T2 imaging and/or mapping	1–3 short-axis views	Myocardial edema
T1 mapping (native, **pre-contrast**)	1–3 short-axis views	Myocardial characterization
Early gadolinium enhancement	Short-axis view	Hyperemia
Late gadolinium enhancement	Short-axis stack Long-axis views	Myocardial inflammation or fibrosis
T1 mapping (**post-contrast**)	1–3 short-axis views LV long-axis view(s)	Myocardial characterization
Additional case-specific or comprehensive imaging		
Myocardial perfusion (with regadenoson **stress** using 1/4 of total gadolinium dose^a^)	1–3 short-axis views LV long-axis view(s)	Coronary artery evaluation
Myocardial perfusion (at **rest** using 1/4 of total gadolinium dose, followed by administration of remainder of gadolinium contrast^a^)	1–3 short-axis views LV long-axis view(s)	Coronary artery evaluation
Dynamic magnetic resonance angiography		Persistent superior cavopulmonary anastomosis assessment
Respiratory navigator-gated, ECG-triggered magnetic resonance contrast angiography		Venous or arterial evaluation
Phase contrast flow	Ascending aorta Main pulmonary artery (if indicated) Atrioventricular valves (if indicated)	Valve dysfunction
Black blood imaging (especially with metallic artifact)	Axial stack	Venous or arterial evaluation

*EF* ejection fraction, *LV* left ventricular, *RV* right ventricular, *bSSFP* balanced steady state free precession

^a^If myocardial perfusion imaging is performed, stress imaging is done after pre-contrast T1 mapping, and rest imaging is done after early gadolinium enhancement

### Cardiac function

Acute and chronic rejection and CAV may result in occult or overt decreased systolic performance. Myocardial mass assessment should be performed at each evaluation. Increases in LV mass may represent true myocardial hypertrophy secondary to systemic hypertension or pseudo-hypertrophy due to acute rejection [164], although this has not been shown in a pediatric population. The first sign of acute or chronic rejection can be development of asymmetric septal hypertrophy, so CMR evaluation should include full 16-segment measurement of wall thicknesses.

### Myocardial characterization

The CMR study for both acute and chronic rejection is based on 3 myocardial characterization techniques. First, T2 imaging or T2 mapping provides good discrimination of normal myocardium from myocardium with increased edema. Edema is a sign of myocardial inflammation that may be a characteristic of either cellular or humoral rejection. In one adult study, a cut-off T2 value of ~ 56 ms was a discriminator below which essentially no biopsies were positive for cellular rejection [165]. Second, T1 values are prolonged in adult transplant recipients with acute rejection [166]. This is true in an exclusively pediatric patient population as well [167]. Third, LGE has shown variable utility in transplant imaging. In most studies LGE is prevalent, but not helpful with acute rejection since it may be related to graft ischemia at the time of transplant or the cumulative result of chronic rejection or coronary allograft vasculopathy [166]. There is a paucity of published pediatric data in this area.

### Coronary artery evaluation

Transplant CAV is a multi-factorial progressive cause of graft failure [168]. Calculating a semi-quantitative myocardial perfusion reserve (MPR) coefficient detects high grade CAV with good sensitivity. An MPR < 1.68 has a high negative predictive value in an adult population, suggesting it would be able to identify patients without significant coronary involvement [169].

### Valve disease

Multiple transvenous biopsies may result in tricuspid valve injuries [170]. Injury to the tricuspid valve chordae or papillary muscles is the most common etiology [171]. Ventricular volumetry combined with phase contrast imaging allows accurate assessment of the tricuspid valve regurgitant fraction. A standard 4-chamber cine acquisition paired with an orthogonal RV inflow-outflow view will depict the location of tricuspid valve dysfunction. Occasionally, the tricuspid regurgitation is severe enough to require surgical therapy, and CMR can be useful for procedural planning.

### Venous or arterial evaluation

Unique to patients transplanted for failed palliation of CHD is the indication for a targeted evaluation of the systemic and pulmonary venous anatomy and the pulmonary and systemic arterial systems [172]. Details depend on the type of palliation and which vessels were involved in the original pathophysiology. Failed palliation of hypoplastic left heart syndrome in particular presents a unique population of transplant patients, requiring evaluation of the reconstructed aorta and the pulmonary arteries. This is in addition to routine surveillance of vascular anastomoses of transplanted hearts.

#### Limitations and pitfalls

There are important limitations to using CMR in transplant patients. A small number of patients have implanted hardware including pacemakers and defibrillators that make CMR a relative contraindication. In older patients transplanted after Fontan palliation, there are often stainless steel vascular occlusion coils that make imaging impossible. Younger patients require either sedation or general anesthesia for a complete CMR with myocardial characterization. There is a small but significant rate of adverse outcomes in heart transplant patients with anesthesia induction only [173]. The sensitivity for both acute rejection and CAV are relatively high using CMR in transplant patients; however, the specificity is low. Consequently, using CMR for excluding the need for biopsy is promising, but differentiating between the grades of low-level rejection or determining the exact sites of low-grade CAV remains elusive. Most of the data in the literature supporting the use of CMR in transplant patients are from small studies without long-term outcome data.

#### Newer techniques

Using myocardial strain assessment for detecting acute rejection has shown promise particularly in the first year after transplantation in an adult population [174]. Although impaired circumferential strain is associated with CAV, it has not proven to be a discriminator for clinically significant CAV [175]. In a small study of 24 children, LGE with vessel wall imaging was shown to correlate with vessel wall thicknesses measured by intravascular ultrasound [176]. The study highlighted the ability of CMR to directly measure vessel wall pathology rather than indirectly assessing the changes caused by CAV.

### Aortopathy and connective tissue disease (CTD)

Aortopathies comprise a heterogeneous group of diseases represented by abnormal configurations of the aorta within the thorax or abdomen. The aortic pathology ranges from focal or global dilatation to focal or global hypoplasia. While this guideline focuses on non-CHD, thoracic aorta dilation associated with bicuspid or unicuspid aortic valves is also addressed given the frequency of this disease. Connective tissue aortopathies in the pediatric population are associated with diseases such as Marfan, Loeys-Dietz, Turner, Noonan, and Ehlers-Danlos syndromes [177–181], which can also be associated with intra-cardiac abnormalities such as mitral valve prolapse. Aortic narrowing can be seen in non-inflammatory diseases such as Williams syndrome. Other congenital aorta anomalies such as vascular rings and abnormal aortic arch branching are not addressed in these guidelines. The inflammatory arterial diseases are covered separately in the KD section above.

While TTE remains the cornerstone of initial pediatric cardiac imaging, even in patients with optimal acoustic windows not all aortic segments can be visualized. For example, the descending thoracic aorta can be masked by ultrasound attenuation artifacts from the mainstem bronchi. In older children, the ascending aorta may not be visualized in its entirety and focal dilatation can remain undetected.

CMR, with its advantage of wide field-of-view imaging, is typically performed when (1) portions of the aorta are not well visualized by TTE, or (2) CMR of the aorta is requested due to the known higher risk for pathology in a given CTD or genetic disease (see below). For example, guidelines for the care of patients with Turner syndrome have advocated using CMR to screen for aortic pathology in all patients old enough to cooperate without sedation [177].

CMR for assessment of the aorta often requires serial scans, such as monitoring for change in an aneurysm at a coarctation repair site or in CTD monitoring for progression of aneurysm. For patients with repaired aneurysm of the aortic root or ascending aorta, published guidelines provide a Class IIa recommendation for serial CT or CMR imaging, preferably with the same modality at the same institution [182]. These authors note that for surveillance of stable and moderate aneurysms, CMR provides adequate information while avoiding repeated radiation exposure from serial CT scanning.

Improvements in non-contrast 3-D imaging allow for some possibility of scanning without contrast. However, contrast-enhanced CMRA continues to provide the best SNR and anatomic detail for assessing the aorta. This is complicated by concern for the repeated use of gadolinium-based contrast agents (GBCA), including evidence for gadolinium deposition in the brain and other tissues [183], including in children [184]. The use of ferumoxytol has emerged as an alternative to GBCA when LGE imaging is not required. This agent is an ultrasmall, superparamagnetic iron oxide particle with strong T1 relaxation effects and a long half-life in the intravascular space [185]. Despite concern and a United States Food and Drug Administration (FDA) black box warning for acute hypersensitivity reactions, particularly with bolus injection for iron deficiency anemia, registry data shows a positive safety profile for the use of feromoxytol in CMR, including in infants and children [186].

#### Goals of examination

Goals of a CMR evaluation can include both anatomic and functional imaging, though the latter is less prevalent in the clinical domain. For anatomic imaging, evaluation of the thoracic aorta should include the aortic valve annulus, sinuses of Valsalva, and sinotubular junction. Depending on the suspected phenotypic or genetic diagnosis, the field of view can be extended to evaluate head and neck vessels, abdominal aorta, or more distal arterial structures. Since branches from the aorta will almost always be included in CMR imaging, these structures should also be evaluated for any focal disease (Table 12).

**Table 12:** Aortopathy and connective tissue disease standard imaging

Sequence	Imaging plane	Indication
Standard imaging		
Cine bSSFP	Parallel to the LVOT in orthogonal planes, short axis of the aortic root, Short-axis of the largest diameter of the ascending aorta, sagittal oblique in long axis of the aortic arch (“candy cane”)	Aortic valve morphology, measurement of aortic root and ascending aorta, and assessment of aortic arch anatomy
Contrast-enhanced magnetic resonance angiography	3D volume, extend field of view superiorly to angle of the jaw	Extracardiac vascular anatomy for aneurysm formation and vertebral tortuosity
3D bSSFP, GRE or mDIXON FSE respiratory navigator-gated and ECG-triggered whole heart sequence^a^	3D volume	Extracardiac vascular assessment for aneurysm formation, measurement of aortic root
Phase contrast flow	Ascending aorta Descending aorta	Flow velocity and pattern
Additional case-specific or comprehensive imaging		
Cine bSSFP	Ventricular short-axis or axial (transverse) stack	LV volumes, mass, and EF RV volumes, mass, and EF
Cine bSSFP	Extend field of view to include head, neck, abdomen, and pelvis	Loeys-Dietz syndrome
Proton-density-weighted FSE black blood imaging	Sagittal oblique in long axis of the aortic arch (“candy cane”)	Vascular anatomy
Cine bSSFP	Short and long axis of the region of interest	Concern for dissection or interarterial thrombus

*3D* 3-dimensional, *ECG* electrocardiogram, *EF* ejection fraction, *GRE* gradient echo, *LV* left ventricular, *RV* right ventricular, *LVOT* left ventricular outflow tract, *bSSFP* balanced steady state free precession, *FSE* fast spin echo

^a^Imaging can be performed during systole or diastole; newer sequences may provide both

Without cardiac gating, the aortic root—including the annulus, sinuses of Valsalva and sinotubular junction—is prone to motion artifacts related to its proximity to the heart. The remainder of the aorta, however, is less susceptible. Contrast-enhanced 3D CMRA of the thoracic aorta and neck allows for measurements of vertebral arterial tortuosity, which has been found to be associated with clinical outcomes in children with CTD [187]. For evaluation of the aortic root, strategies to reduce pulsatility-related image degradation should be considered, including cardiac gating using ECG-gated 2D cine bSSFP or ECG-gated and respiratory navigated 3D bright blood imaging in systole. ECG-gated cine bSSFP imaging is also used to assess mitral valve pathology associated with CTD such as prolapse and mitral annular disjunction.

In addition to the vascular system, other regions of the body can be evaluated for signs that would suggest a specific connective tissue etiology and diagnosis. In Marfan syndrome, for example, consideration can be given for the evaluation of non-vascular structures such as spine imaging for dural ectasia [181]. The geometry of aneurysms should be detailed and can involve additional long, short, or other oblique planes. Although not a typical indication for pediatric patients, in hemodynamically stable patients CMR can be employed to look for markers of aortic dissection [188].

For functional imaging, phase contrast pulse sequences with through-plane velocity encoding can assess ascending aorta and descending aorta flow. Velocity encoding selection will be highly variable and based on the type of pathology. Comparison of forward flow through the mitral valve with net aortic valve flow yields a mitral regurgitant fraction in the anatomically normal heart with CTD and mitral valve pathology.

#### Measurement guidelines

**Fig. 6:**
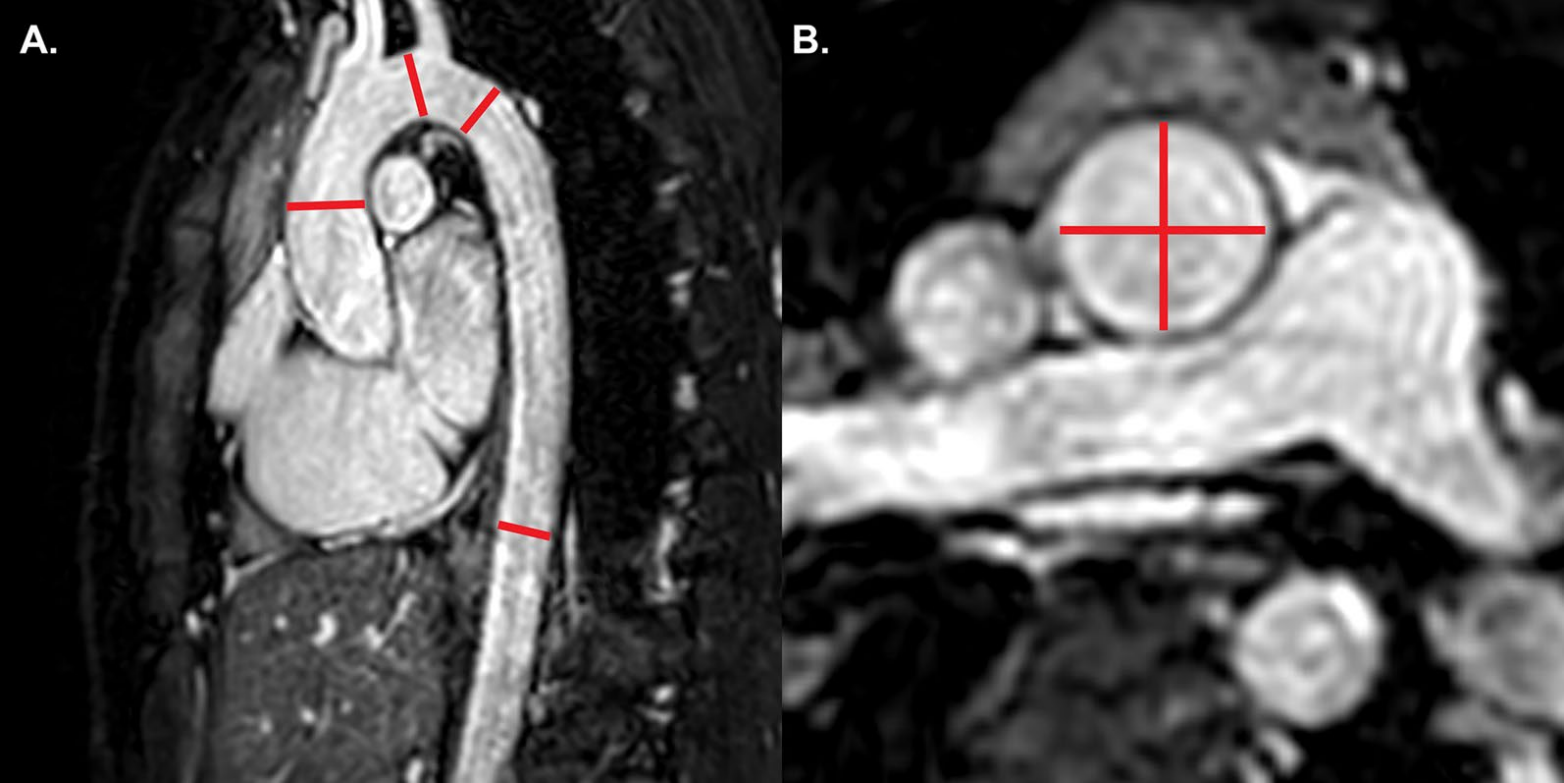
Aortic measurements at multiple levels. 3D bSSFP image, reconstructed into the long axis of the aortic arch (**A**). Lines display recommended locations for measurement: ascending aorta, distal transverse aortic arch, aortic isthmus and descending aorta at the diaphragm. Double oblique images should be reconstructed at each of these levels (**B**-ascending aorta) for en face measurements

There are several pulse sequences that can be used to image and characterize the aorta, with important differences between sequences that may affect measurements, such as the presence or absence of cardiac gating. Contrast-enhanced CMRA, for example, is typically performed without ECG gating, meaning that vessel measurements are averaged through the cardiac cycle. Given this possible variation, it is recommended that both imaging and measurement protocols are standardized within an institution. Measurements should be made in a cross-sectional plane to the aorta, inner edge to inner edge, using reconstructed 3D data or double oblique cine bSSFP sequences for systolic measurements. Locations of which segments of the thoracic aorta are to be measured have been published in adult guidelines [189, 190] and can be adapted for pediatric patients (Fig. 6). The report itself and/or secondary image captures should document (1) the specific sequences that were used for aortic measurements; (2) phase in the cardiac and respiratory cycles if applicable; and (3) consistent window width and level selections, which ideally should be similar between serial measurements. Aortic measurements should be reported separately from the text of the findings in clearly marked fields.

**Fig. 7:**
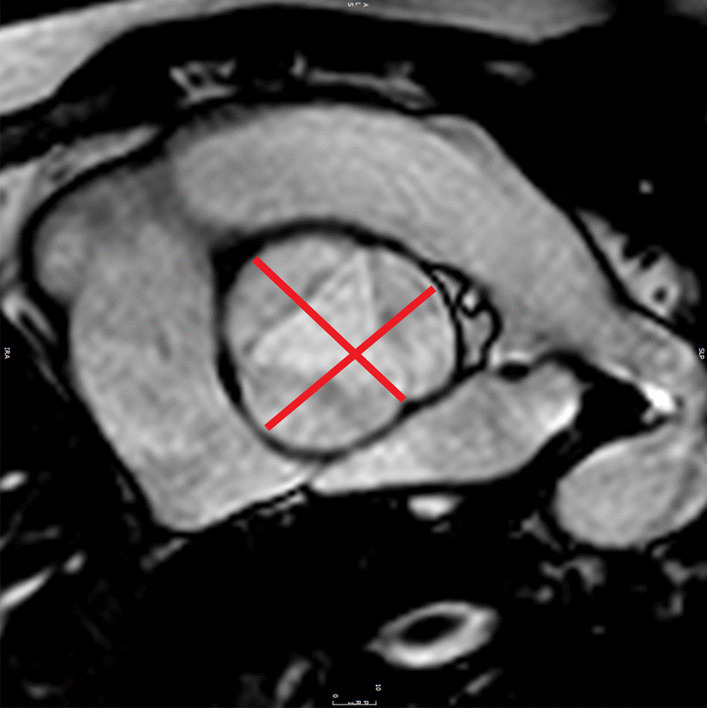
Measurement of the aortic root at the sinuses of Valsalva. Systolic frame of a cine bSSFP image in the short axis of the aortic root, at the level of the sinuses of Valsalva. Lines display recommended measurements, inner edge to inner edge at the largest sinus-to-sinus dimension and the largest commissure-to-sinus dimension

Measurement of the sinuses of Valsalva (aortic root) is particularly complex. The geometry can be asymmetric, especially in patients with dilation and abnormal aortic valve morphology. Because of this geometric complexity, selection and measurement of the largest dimensions within the aortic root may be best determined using double oblique planes derived from 3D data sets. This may also provide better reproducibility by allowing side-by-side reconstruction of 3D data from sequential studies. We recommend measuring the aortic root at the sinuses of Valsalva en face in systole, either from cine bSSFP images in the short-axis of the aortic root, or from a reconstructed 3D, cardiac gated dataset, acquired in systole. Recommended measurements include (1) the largest sinus to sinus diameter and (2) the largest commissure to sinus diameter (Fig. 7). An additional measurement of the maximal sinus diameter from a 3-chamber view is optional, with the advantage of measurement in a similar plane to that made by echocardiography. Some centers additionally measure the cross-sectional area of the aortic root; while there are currently no normative data, and no data linking to clinical outcomes, this measurement could prove to be meaningful and reproducible. End-systolic measurements are recommended to obtain the maximal dimension of the aorta and so that CMR measurements will correlate better with pediatric TTE findings [191]; the pediatric guidelines contrast with adult echocardiography guidelines which recommend measurement in diastole [190].

#### Limitations and pitfalls

Implants such as spinal rods or other metallic items may limit the quality of aortic imaging. While normal data for pediatric aorta sizes have been published [192], these are limited by small sample sizes, especially when factoring in small patients such as infants and toddlers. In addition, there are no published normal data that factor for potential differences due to the varied sequences with which the aorta may be imaged.

#### Newer techniques

**Fig. 8:**
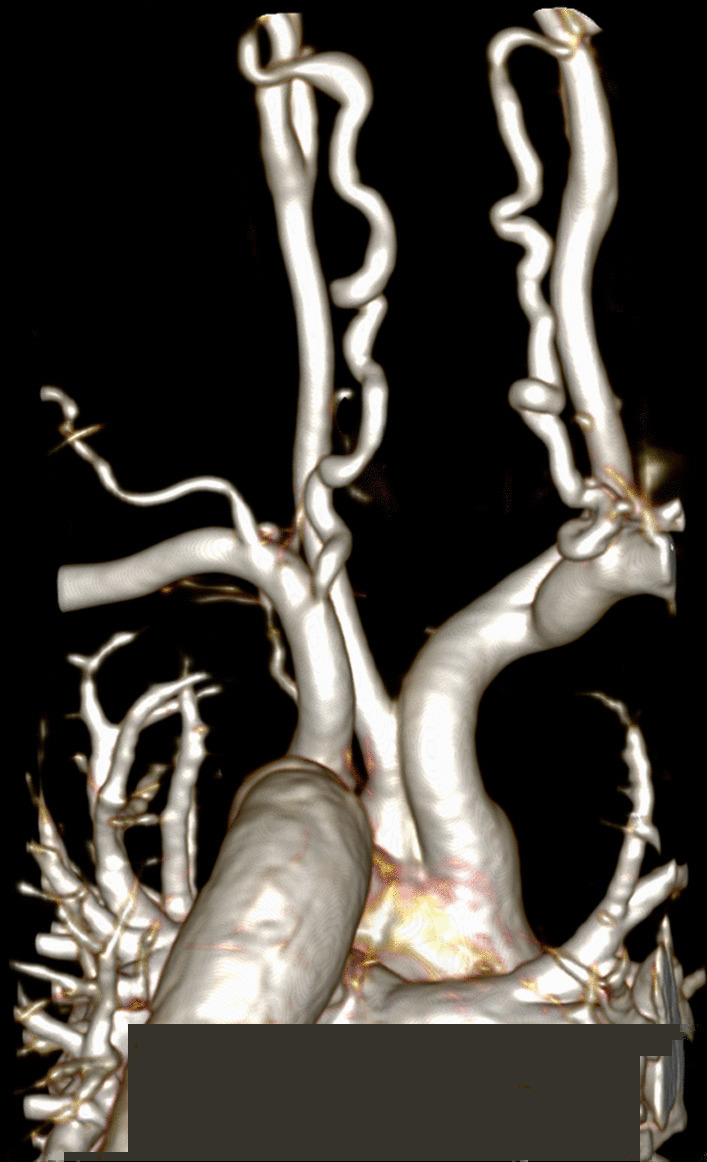
Tortuous vertebral arteries in connective tissue disease. 3D reconstruction of a gadolinium-enhanced cardiovascular magnetic resonance angiogram (CMRA), showing tortuous vertebral arteries in a patient with Marfan syndrome. This image can be used to calculate the vertebral tortuosity index

While standard clinical imaging focuses on the dimensions and geometry of the thoracic aorta, vascular assessment can include 4D flow, head and neck vessel tortuosity (Fig. 8) [187], arterial wall anatomy, endothelial function, and mechanical property evaluation [193]. Measurement of the vertebral tortuosity index [187] should be strongly considered in patients with CTD and requires extending the field of view of the CMRA superiorly to the angle of the jaw. Techniques that can evaluate arterial mechanical properties include CMR indices of aortic stiffness, pulse wave propagation velocities, and afterload-effects from pulse wave reflections. Many of these assessments in pediatric patients remain investigational.

### Structured CMR reporting in pediatric patients

The reporting of CMR results in children should conform to the structured format seen in other pediatric cardiac imaging modalities. The standard pediatric CMR report can be divided into 6 key sections: (1) Administrative, (2) Patient Demographics, (3) Study Performance Information, (4) Structured Cardiovascular Findings, (5) Measurements, and (6) Summary of Findings. The SCMR has propagated guidelines for the standard CMR report [194], with further guidance provided by the Canadian SCMR [38]. Although details of the structured report vary, there is general agreement on most of the required elements recommended in Table 13.

**Table 13:** Structured cardiac magnetic resonance examination reporting elements

Administrative	
Recommended	Site of service Scanner field strength and model Procedure date and time Referring physician Referring clinical information, including indication(s)
Optional	Laboratory accreditation status
Patient demographics	
Recommended	Unique patient identification number Date of birth Gender Height, weight, and body surface area
Optional	Race and ethnicity Heart rate Blood pressure Renal function assessment
Study performance	
Recommended	Description of the study, including sequences Contrast agent, if any Sedation or anesthesia, if any Significant study limitations, if any Adverse events, if any
Optional	Personnel involved in the procedure
Structured cardiovascular findings	
Recommended	Segmental diagnoses Ventricular volume, function, and mass assessment Regional wall motion assessment, if appropriate Late gadolinium enhancement, if appropriate
Optional/as applicable	Significant non-cardiovascular findings, if any Key images
Measurements	
Recommended	Biventricular volume, function, and left ventricular mass measurements
Optional/as applicable	Phase contrast flow measurements of the ascending aorta, main and branch pulmonary arteries Aortic measurements (with normal range as available)
Summary of findings	
Recommended	Abnormal findings Pertinent negative findings Comparison with previous studies, if appropriate
Optional	Differential diagnoses Recommendations for further imaging

The terminology used in the structured CMR report should be simple and conform to the recommendations of professional societies [195]. Cardiovascular measurements should be adjusted for body size, and normal ranges should be provided, with citations where appropriate. The CMR sequence from which the measurements were made should be listed. The effective CMR examination report will provide a detailed list of pertinent findings [1, 38], including an overall impression with supporting evidence [196, 197].

From the recommendations above, the structured CMR report should include several items specific to pediatric patients. The potential impact of sedation on findings may require special notation. The examination and reporting of the aortic root in pediatric studies for aortopathy merits special mention, and this issue was discussed extensively in this document. Of note, these recommendations differ significantly from the standard adult CMR protocols [2].

The techniques utilized to measure cardiovascular dimensions, volumes, and mass should conform to the techniques used in the studies cited for normal values. When appropriate, the techniques utilized and any limitations of normal values for the youngest children should be noted. There will be many instances—such as with cardiomyopathies, aortopathy, or pulmonary hypertension—when CHD co-exists with an acquired lesion, and examination protocols and reporting should be adjusted accordingly.

## Data Availability

Data sharing is not applicable to this article as no datasets were generated or analyzed during the current study.

## Abbreviations

3D: Three dimensional
4D: Four dimensional
AC: Arrhythmic cardiomyopathy
ARVC: Arrhythmogenic right ventricular cardiomyopathy
BSA: Body surface area
bSSFP: Balanced steady state free precession
C: Compacted
CAA: Coronary artery aneurysm
CAV: Coronary artery allograft vasculopathy
CCMRA: Coronary cardiovascular magnetic resonance angiography
CHD: Congenital heart disease
CMR: Cardiovascular magnetic resonance
CMRA: Cardiovascular magnetic resonance angiography
COVID-19: Coronavirus disease 2019
CT: Computed tomography
CTD: Connective tissue disease
DCM: Dilated cardiomyopathy
ECG: Electrocardiogram
ECV: Extracellular volume fraction
EF: Ejection fraction
EMB: Endomyocardial biopsy
FT: Feature tracking
GBCA: Gadolinium-based contrast agents
GCS: Global circumferential strain
GLS: Global longitudinal strain
GRE: Gradient echo
GRS: Global radial strain
HCM: Hypertrophic cardiomyopathy
HF: Heart failure
KD: Kawasaki disease
LA: Left atrium/left atrial
LGE: Late gadolinium enhancement
LV: Left ventricle/left ventricular
LVEDVI: Left ventricular end-diastolic volume indexed to body surface area
LVEF: Left ventricular ejection fraction
LVNC: Left ventricular non-compaction
LVOT: Left ventricular outflow tract
MIS-C: Multisystem inflammatory syndrome in children
MOLLI: Modified Look-Locker inversion recovery
MPR: Myocardial perfusion reserve
NC: Non-compacted
NPO: Nil per os
PH: Pulmonary hypertension
Qp:Qs: Ratio of pulmonic flow to systemic flow
RA: Right atrium/right atrial
RV: Right ventricle/right ventricular
RVEF: Right ventricular ejection fraction
RVOT: Right ventricular outflow tract
SASHA: Saturation recovery single shot acquisition
SCMR: Society for Cardiovascular Magnetic Resonance
SENC: Sensitivity encoding
SNR: Signal-to-noise ratio
SPAMM: Spatial modulation of magnetization
STIR: Short tau inversion recovery
TR: Repetition time
TTE: Transthoracic echocardiography
